# The Transcription Factors Sox10 and Myrf Define an Essential Regulatory Network Module in Differentiating Oligodendrocytes

**DOI:** 10.1371/journal.pgen.1003907

**Published:** 2013-10-31

**Authors:** Julia Hornig, Franziska Fröb, Michael R. Vogl, Irm Hermans-Borgmeyer, Ernst R. Tamm, Michael Wegner

**Affiliations:** 1 Institut für Biochemie, Emil-Fischer-Zentrum, Universität Erlangen-Nürnberg, Erlangen, Germany; 2 ZMNH, Universitätsklinikum Eppendorf, Hamburg, Germany; 3 Institut für Humananatomie und Embryologie, Universität Regensburg, Regensburg, Germany; Stanford University School of Medicine, United States of America

## Abstract

Myelin is essential for rapid saltatory conduction and is produced by Schwann cells in the peripheral nervous system and oligodendrocytes in the central nervous system. In both cell types the transcription factor Sox10 is an essential component of the myelin-specific regulatory network. Here we identify Myrf as an oligodendrocyte-specific target of Sox10 and map a Sox10 responsive enhancer to an evolutionarily conserved element in intron 1 of the *Myrf* gene. Once induced, Myrf cooperates with Sox10 to implement the myelination program as evident from the physical interaction between both proteins and the synergistic activation of several myelin-specific genes. This is strongly reminiscent of the situation in Schwann cells where Sox10 first induces and then cooperates with Krox20 during myelination. Our analyses indicate that the regulatory network for myelination in oligodendrocytes is organized along similar general principles as the one in Schwann cells, but is differentially implemented.

## Author Summary

In recent years it has become clear that complex developmental processes are not regulated by single transcription factors but rather by combinations of transcription factors that interact in intricate regulatory networks. Here, we analyze the regulatory network that drives terminal differentiation of oligodendrocytes, the cells of the vertebrate central nervous system that form myelin and thereby guarantee rapid saltatory conduction. We show that the transcription factor Myrf is directly activated by the transcription factor Sox10, and map a Sox10-responsive enhancer to an evolutionarily conserved element in intron 1 of the *Myrf* gene. We then go on to show that once induced, Myrf physically interacts and functionally cooperates with its inducer Sox10 to activate myelin genes arguing that the two jointly drive terminal differentiation of oligodendrocytes. With this study we define an essential module in the myelin-specific regulatory network in the central nervous system. By comparing this module with the corresponding module in Schwann cells of the peripheral nervous system which consists of Sox10 and the Krox20 transcription factor we furthermore conclude that myelination in the two compartments of the vertebrate nervous system is regulated by similarly organized, but differentially implemented regulatory networks.

## Introduction

Myelin is a vertebrate invention and provides the basis for rapid saltatory conduction throughout the nervous system. It is produced by oligodendrocytes (OL) in the central nervous system (CNS) and Schwann cells in the peripheral nervous system (PNS) during terminal differentiation. In these cells myelin formation is controlled by a specialized regulatory network that – like myelin - is restricted to vertebrates.

Much has been learned about this network in myelinating Schwann cells in which the transcription factor Sox10 plays a central role (for review, see [1]). Sox10 is required during all developmental stages and causes the sequential induction of stage-specific transcription factors that cooperate with Sox10 in the next stage of development [2]–[4]. In pro-myelinating Schwann cells Sox10 teams up with its target Oct6 [5] to induce the zinc finger transcription factor Krox20 [6]. This sets the stage for terminal differentiation as the combination of Sox10 and Krox20 constitutes the main driving force for PNS myelination [7], [8].

Transcription factors with roles in differentiation and CNS myelination have also been identified in OL (for review, see [9]). Several key factors such as Olig2, Olig1 and Nkx2.2 differ from the ones known to be important in Schwann cells [10]–[12], and only few interactions have been defined so far [13], [14]. As a consequence, it is not yet clear how these OL-specific transcription factors cooperate in their regulatory network to achieve myelination.

One of the transcription factors with relevance for Schwann cell and OL differentiation is Sox10. It therefore provides an excellent tool to study the myelin-specific regulatory network in both glial cell types. In the oligodendroglial lineage Sox10 expression starts early in the oligodendrocyte precursor cell (OPC) where it is already required in combination with its close relative Sox9 for survival and proper migration [15]–[17]. Its most obvious function, however, is during terminal differentiation and myelination. At the time of birth Sox10-deficient mice possess a full complement of OPC, yet exhibit little myelin gene expression [16]. This is in part attributed to direct activation of myelin genes by Sox10 [16], [18].

Myelin Regulatory Factor (Myrf, also known as Mrf and Gm98) is another transcription factor critically required for CNS myelination [19]. Unlike Sox10, Myrf is not expressed in OPC and becomes expressed only upon cell cycle exit in promyelinating OL. Constitutive Myrf-deficient mice exhibit severe myelination defects. When *Myrf* is deleted conditionally in mature OL, oligodendroglial identity and the integrity of CNS myelin cannot be maintained [20]. Whereas myelin gene expression is strongly dependent on Myrf, Sox10 levels are not [19].

Considering the involvement of Sox10 and Myrf in OL differentiation and myelination, their genetic and functional relationship needs to be determined. As expression patterns and published evidence argue against a role for Myrf in Sox10 induction, we analyzed whether Sox10 is required for Myrf expression using mice in which Sox10 was selectively deleted in the CNS. These mice suffered from severe hypomyelination, but survived for three weeks after birth. Their analysis and accompanying studies show that Myrf is not only genetically downstream of Sox10, but also represents a direct Sox10 target gene. Once induced by Sox10, Myrf cooperates with its inducer to jointly activate myelination. Its function and relation to Sox10 is thus similar to Krox20 in Schwann cells. The present study defines a key circuit of the myelin-specific regulatory network in OL and emphasizes similarities and differences to Schwann cells.

## Results

### CNS-Specific Sox10 Deletion Results in Severe Myelination Defects and Death during the First Month of Life

Mice with constitutive Sox10 deletion are unable to breathe because of severe PNS defects and die immediately after birth [2]. At this time, OL differentiation and CNS myelination have barely started so that these mice do not allow a full evaluation of Sox10 in CNS myelination [16]. To sidestep perinatal lethality, we generated mice in which Sox10 was specifically deleted in the CNS using a floxed *Sox10* allele [3] in combination with a *Brn4::Cre* transgene [21]. The resulting mice are henceforth referred to as *Sox10^ΔCNS^*. In these mice Cre expression is restricted to CNS and limb bud ectoderm. Within the CNS, Cre is already present throughout the early ventricular zone and allows efficient Cre-mediated gene deletion in neural precursors and all neurons and glia derived from them.

On a gross morphological level, *Sox10^ΔCNS^* mice were indistinguishable from littermates during the first days after birth. However, starting from the second week *Sox10^ΔCNS^* pups shivered and exhibited an unsteady, shaky gait. Most *Sox10^ΔCNS^* mice died at the end of the third week. None survived past postnatal day (P) 24.

For analysis we concentrated on the spinal cord. In *Sox10^ΔCNS^* mice, *Sox10* was already efficiently deleted by 12.5 days post coitum (dpc) so that OPC developed in the complete absence of Sox10 (data not shown). As a consequence and in contrast to the wildtype, Sox10 was absent from the postnatal spinal cord of mutant mice (compare Figure S1A–D,S to Figure S1E–H,S). Sox10 deletion did not dramatically alter oligodendroglial expression of its close relatives Sox8 and Sox9 (Figure S1I–R). Sox9 was equally down-regulated in wildtype and *Sox10^ΔCNS^* spinal cord during the phase of active myelination (Figure S1T). Sox8 remained expressed despite the absence of Sox10 (Figure S1M–P). The fact that there were fewer Sox8-positive cells from P7 onwards (Figure S1U) reflects the reduced number of oligodendroglial cells in the mutant (see below).

To further characterize the phenotype of *Sox10^ΔCNS^* mice, we analyzed oligodendroglial lineage and differentiation markers including the OPC marker Pdgfra. At 18.5 dpc, the number of *Pdgfra*-positive OPC was very similar in wildtype and *Sox10^ΔCNS^* embryos as determined by in situ hybridization (Figure S2A–C). This agreed well with previous findings on *Sox10^−/−^* mice [16] and argues that OPC numbers and - because they make up the vast majority of oligodendroglial cells in the late prenatal spinal cord - overall oligodendroglial cell numbers are not affected during embryogenesis.

Extending the studies to the early postnatal period, we performed immunohistochemical analyses for the lineage marker Olig2 (Figure S2D–L). At P3, the number of Olig2-positive cells was as high in *Sox10^ΔCNS^* spinal cord as in wildtype (Figure S2D,E,I) again confirming that oligodendroglial cell numbers are first not affected by Sox10 loss. From P3 to P7, the number of Olig2-positive cells further increased in the wildtype spinal cord before reaching its maximum at the end of the second week (Figure S2D,F–H). This increase was not observed in *Sox10^ΔCNS^* mice (Figure S2D,J–L). Instead, the number of Olig2-positive cells remained fairly constant in *Sox10^ΔCNS^* mice over the first three postnatal weeks.

**Figure 1:**
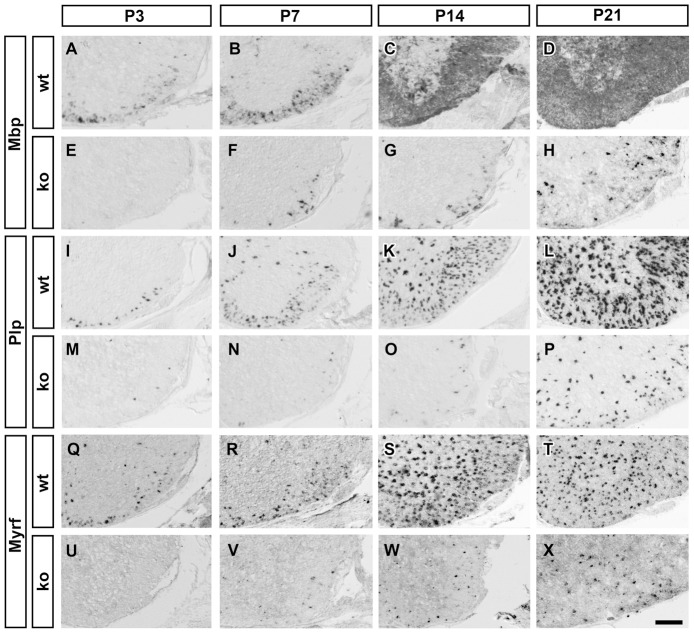
Consequences of CNS-specific Sox10 deletion on the expression of myelination-associated genes in OL. Differentiating OL were visualized by in situ hybridization on transverse spinal cord sections from the forelimb region of wildtype (wt) (**A–D,I–L,Q–T**) or *Sox10^ΔCNS^* (ko) (**E–H,M–P,U–X**) mice at P3 (**A,E,I,M,Q,U**), P7 (**B,F,J,N,R,V**), P14 (**C,G,K,O,S,W**) and P21 (**D,H,L,P,T,X**) using antisense probes against *Mbp* (**A–H**), *Plp* (**I–P**), and *Myrf* (**Q–X**). Ventral horn region is shown. Scale bar, 200 µm.

**Figure 2:**
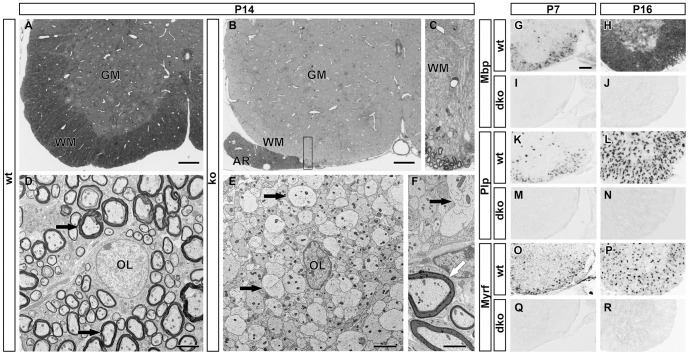
Histological analysis of myelination after CNS-specific Sox10 deletion and consequences of combined deletion of Sox8 and Sox10 on myelin gene expression. (**A–C**) Light microscopy of 1 µm semi-thin sections of the spinal cord ventral horn in wildtype (wt) (**A**) and *Sox10^ΔCNS^* (ko) mice (**B,C**) at P14 following Richardson's stain. Myelinated axons are stained in the white matter (WM) of the wildtype, but not the mutant. Sections from *Sox10^ΔCNS^* mice contain myelin only in the anterior rootlet (AR) where it is formed by Schwann cells. **C** represents a higher magnification of the area boxed in B. GM, grey matter. Scale bars, 100 µm. (**D–F**) Transmission electron microscopy of the wildtype spinal cord ventral horn at P14 shows myelinated axons (**D**, black arrows) around an OL (OL) in the white matter. In contrast, OL in *Sox10^ΔCNS^* mice are surrounded by axons that lack myelin sheaths (**E**,**F**, black arrows). In mutant mice only Schwann cells in the anterior rootlet have formed myelin sheaths (**F**, white arrow). Scale bars, 2.5 µm. (**G–R**) Differentiating OL were visualized by in situ hybridization on transverse spinal cord sections from the forelimb region of wildtype (wt) (**G,H,K,L,O,P**) or *Sox10^ΔCNS^ Sox8^lacZ/lacZ^* (dko) (**I,J,M,N,Q,R**) mice at P7 (**G,I,K,M,O,Q**), and P16 (**H,J,L,N,P,R**) using antisense probes against *Mbp* (**G–J**), *Plp* (**K–N**), and *Myrf* (**O–R**). Ventral horn region is shown. Scale bar, 200 µm.

Myelin gene expression in the postnatal *Sox10^ΔCNS^* spinal cord was analyzed by in situ hybridization with *Mbp* and *Plp* probes. At P3, *Mbp*- or *Plp*-positive cells were rarely seen in the mutant white matter, while present in substantial numbers in the wildtype (compare Figure 1A,I to Figure 1E,M). At P7, *Mbp*- and *Plp*-positive cells had increased in the *Sox10^ΔCNS^* spinal cord, but were still much fewer than in the wildtype (compare Figure 1B,J to Figure 1F,N). This trend persisted: The number of *Mbp*- and *Plp*-expressing cells in *Sox10^ΔCNS^* mice continued to increase, but their number failed to catch up with the wildtype until time of death (compare Figure 1C,D,K,L to Figure 1G,H,O,P). Histological and ultrastructural analyses of spinal cord white matter from *Sox10^ΔCNS^* mice at P14 failed to detect myelinated axons (compare Figure 2A with Figure 2B, Figure 2D with Figure 2E). The only myelin present in *Sox10^ΔCNS^* mice was in PNS structures such as spinal nerve rootlets and produced by Sox10-expressing Schwann cells (Figure 2B,C,F).

### Myelin Gene Expression Is Completely Blocked in *Sox10^ΔCNS^* Mice upon Additional Sox8 Deletion

Despite complete loss of myelin, residual myelin gene expression was still observed in *Sox10^ΔCNS^* mice. We have previously shown that the closely related Sox8 is co-expressed with Sox10 during OL development and participates in OL differentiation, although it cannot replace Sox10 or fully compensate its loss [22], [23]. To test whether Sox8 is responsible for the residual myelin gene expression in *Sox10^ΔCNS^* mice we generated *Sox10^ΔCNS^ Sox8^lacZ/lacZ^* mice, and analyzed myelin gene expression until time of death during the third postnatal week. After combined loss of Sox8 and Sox10, *Mbp* and *Plp* expression was completely missing (compare Figure 2G,H,K,L to Figure 2.I,J,M,N). Immunohistochemistry for Olig2 on P7 confirmed that Olig2-positive cells were still present, although in slightly reduced number (29.8±4.0% of spinal cord cells in *Sox10^ΔCNS^ Sox8^lacZ/lacZ^* mice as compared to 36.5±1.6% in the wildtype, corresponding to a reduction of 18% in the mutant). This confirms that residual myelin gene expression in *Sox10^ΔCNS^* mice is Sox8-dependent.

### OL Development Is Already Altered at the Late Promyelinating Stage in the Absence of Sox10

In situ hybridizations were also carried out with a *Myrf* probe. Intriguingly, *Myrf* expression was dramatically reduced in *Sox10^ΔCNS^* mice. *Myrf*-positive cells appeared in substantial numbers not before P7 and increased slowly until P21 without ever coming close to wildtype numbers (compare Figure 1Q–T to Figure 1U–X). Upon additional deletion of Sox8, *Myrf* expression was completely absent at all times (compare Figure 2O,P to Figure 2Q,R).

**Figure 3:**
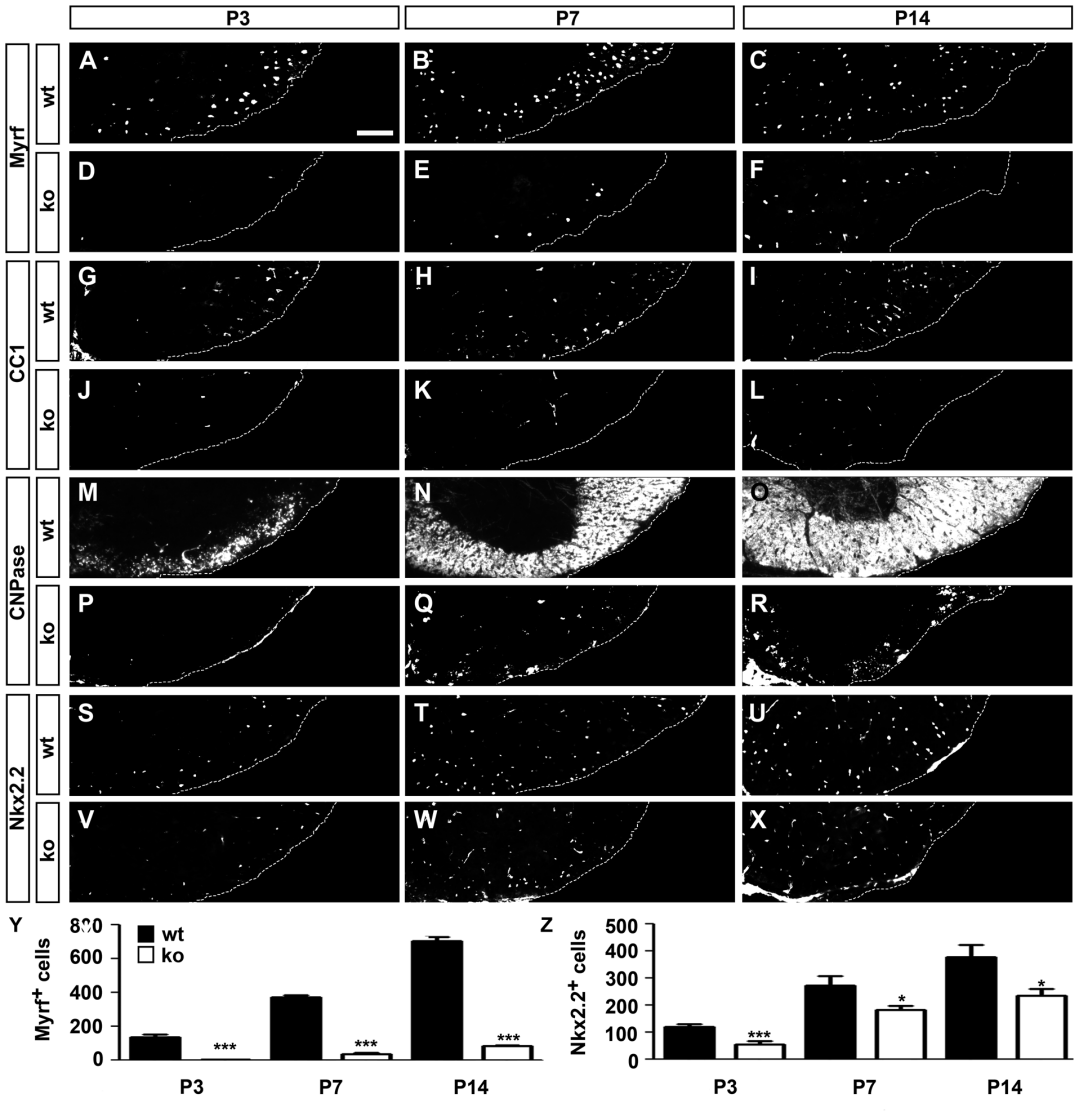
Consequences of CNS-specific Sox10 deletion on the expression of marker proteins of differentiating OL. (**A–X**) Immunohistochemistry was performed on transverse spinal cord sections from the forelimb region of wildtype (wt) (**A–C,G–I,M–O,S–U**) or *Sox10^ΔCNS^* (ko) (**D–F,J–L,P–R,V–X**) mice at P3 (**A,D,G,J,M,P,S,V**), P7 (**B,E,H,K,N,Q,T,W**) and P14 (**C,F,I,L,O,R,U,X**) using antibodies directed against Myrf (**A–F**), CC1 (**G–L**), CNPase (**M–R**), and Nkx2.2 (**S–X**). Ventral horn region is shown. Scale bar, 75 µm. (**Y,Z**) From these stainings, the total number of Myrf-positive (**Y**) and Nkx2.2-positive cells in the white matter (**Z**) was quantified in wildtype (black bars) and *Sox10^ΔCNS^* (white bars) mice. At least 9 separate sections from the forelimb region of 3 independent specimens were counted for each age and genotype. Data are presented as mean ± SEM for biological replicates. Differences to the wildtype were statistically significant for oligodendroglial cell numbers between wildtype and mutant from P3 onwards as determined by the Student's *t* test (*, P≤0.05; ***, P≤0.001).

The reduction in Myrf expression was not only observed on transcript but also on protein level (compare Figure 3A–C to Figure 3D–F, and for quantitation Figure 3Y), and resembled the markedly reduced expression of the early oligodendroglial differentiation marker CC1 (Figure 3G–L). Expression of 2′,3′-cyclic nucleotide 3′ phosphodiesterase (CNPase) was drastically reduced as well (Figure 3M–R). Two markers of the promyelinating stage, in contrast, exhibited only mild (Nkx2.2) or no (Gpr17) alterations in *Sox10^ΔCNS^* mice (Figure 3S-X-,Z and Figure S3A).

RT-PCR experiments on RNA from P7 spinal cord confirmed a statistically significant downregulation of *Mbp* and *Myrf* (Figure S3B). *Zfp488* as a transcriptional regulator in already myelinating OL [24] was similarly downregulated, whereas *Nkx2.2* expression was much less affected in good agreement with the immunohistochemical data. No alterations in transcript levels were observed for *Olig2*, *Pdgfra*, *Zfp191*, *Tcf4*, *Yy1*, and *Hes5* in P7 spinal cord of Sox10^ΔCNS^ mice relative to wildtype mice, while there was a trend towards upregulation for *Id2* (P = 0,09) and statistically significant upregulation for *Id4* in the mutant (Figure S3B). Similar results were also obtained at P14 – the main differences being that at this later stage *Olig2* and *Nkx2.2* levels were also significantly downregulated and levels of *Id2* and *Id4* were no longer increased (Figure S3C). Considering that most OPC markers, and Nkx2.2 and Gpr17 as two markers of the promyelinating stage exhibited only mild changes in their expression, while markers of the myelinating stage were severely affected, it seems reasonable to assume that oligodendroglial development in *Sox10^ΔCNS^* mice is blocked at the transition from the promyelinating into the myelinating stage. Myrf appears to be one of the earliest, severely affected markers.

### Myrf Is a Direct Target of Sox10

**Figure 4:**
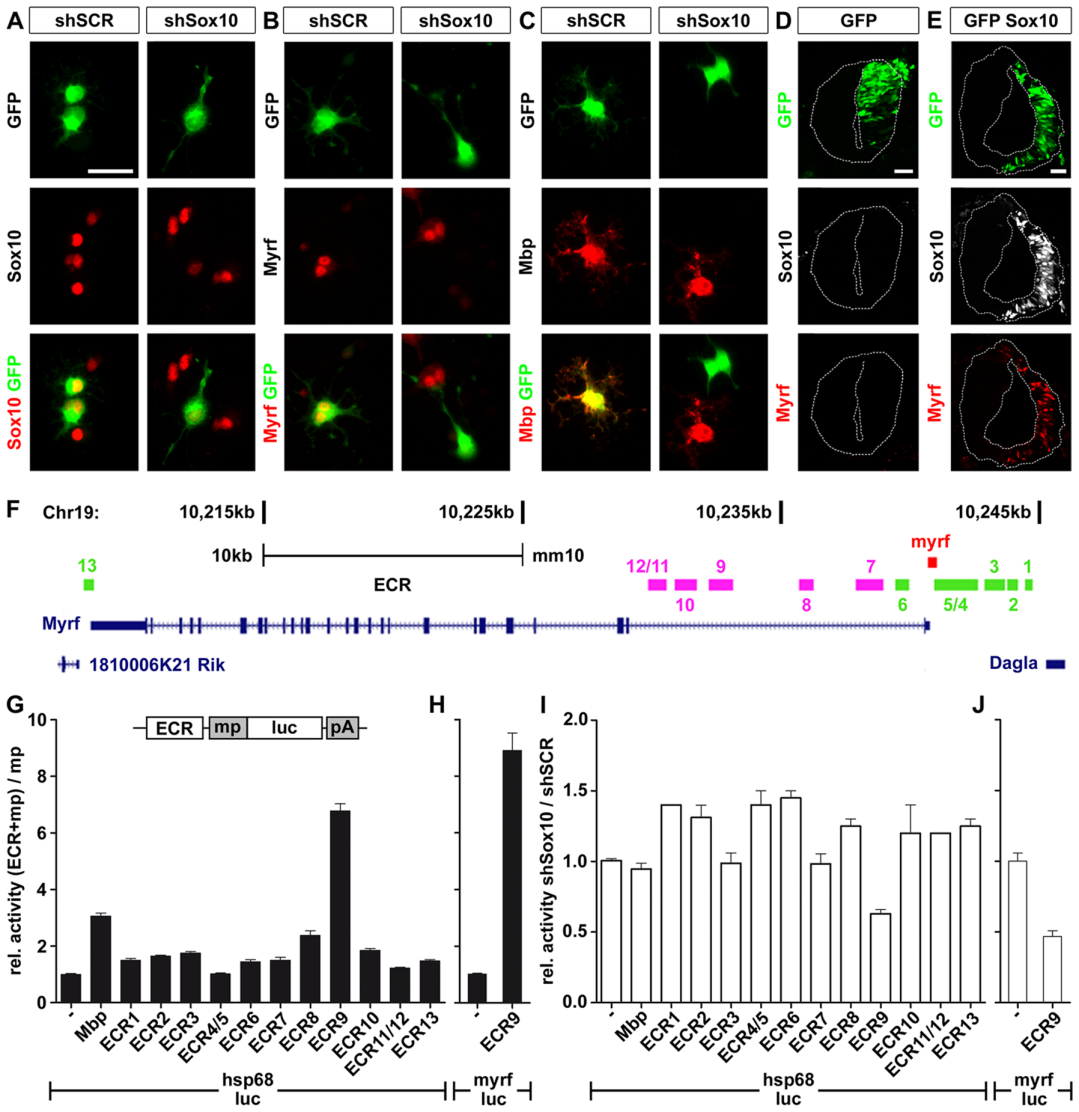
Myrf is a Sox10 target gene in OL. (**A–C**) Primary rat OPC were transfected with expression vectors for scrambled (shSCR) or Sox10-specific shRNAs (shSox10) and GFP, and replated in differentiation medium. One day later, transfected cells were visualized by GFP expression (green) and analyzed for their expression of Sox10 (**A**), Myrf (**B**) and Mbp (**C**) (all in red) as indicated. The yellow color in the merged pictures indicates co-expression. Scale bar, 25 µm. (**D,E**) Neural tube electroporations were carried out in HH11-stage chicken embryos using expression vectors for GFP (**D**) and a combination of Sox10 and GFP (**E**). Analysis was one day after electroporation and the electroporated right side is visualized by GFP expression (green). Sections were simultaneously probed for the occurrence of Sox10 (white) and Myrf (red). Scale bars, 25 µm. (**F**) Several ECR (*ECR1-ECR13*) are localized in the *Myrf* genomic interval on mouse chromosome 19 between the adjacent *Dagla* and *1810006K21 Rik* genes. ECR locations relative to introns and exons of the *Myrf* gene are shown. *ECR1-ECR6* and *ECR13* (marked in green) are conserved among mammals, *ECR7-ECR12* (marked in pink) additionally in birds. (**G, H**) The *Myrf* ECR were tested in 33B cells after transient transfection for their ability to increase expression of a luciferase reporter under control of a minimal promoter (mp). The minimal promoter was taken from the *Hsp68* gene (*hsp68-luc*) (**G**) or the *Myrf* gene (*myrf-luc*) (**H**). Luciferase activities in extracts from transfected cells were determined 48 hours post-transfection in three experiments each performed in quadruplicates. The luciferase activity obtained for a reporter plasmid containing only the minimal promoter (−) was arbitrarily set to 1. Activities in the presence of ECRs were calculated relative to minimal promoter activity and are presented as mean ± SEM. A reporter in which the minimal promoter was combined with *Mbp* regulatory regions served as positive control. (**I, J**) Transfections of the ECR containing *hsp68-luc* (**I**) and *myrf-luc* (**J**) reporters were carried out in the presence of Sox10-specific shRNA (shSox10) and scrambled (shSCR) shRNA. Luciferase activities were determined and the ratio of activities in the presence of Sox10-specific shRNA versus scrambled shRNA was calculated. Normalized values are presented as mean ± SEM. Experiments were performed three times in quadruplicates. shSox10-dependent downregulation of the activity of ECR9-containing luciferase reporters was statistically significant (P≤0.05, determined by Student's *t* test).

To explore the relationship between Sox10 and Myrf, we first used primary oligodendroglial cultures. We transfected OPC with a Sox10-specific shRNA or a scrambled version in the presence of GFP, and analyzed the cells after one day in culture under differentiating conditions. While nearly all cells (98±2%) transfected with a scrambled shRNA exhibited Sox10 expression, only very few (16±5%) continued to be Sox10-positive in the presence of a Sox10-specific shRNA (Figure 4A). At the same time, cells transfected with the Sox10-specific shRNA failed to induce Myrf or Mbp expression in contrast to untransfected cells and cells transfected with the scrambled shRNA version (Figure 4B,C). This confirms that Myrf expression depends on Sox10.

To complement the loss-of-function studies in *Sox10^ΔCNS^* mice and oligodendroglial cultures, we performed gain-of-function studies in the early chicken embryo (Figure 4D,E). We electroporated Sox10 into the neural tube of chicken embryos at Hamburger-Hamilton (HH) stage 11. 24 h later, we detected Myrf on the electroporated side of the neural tube but not on the non-electroporated side (Figure 4E) or in control neural tubes that were electroporated with GFP only (Figure 4D). Myrf induction occurred only in cells with very high levels of Sox10 whereas induction of the neural crest marker HNK-1 was seen under identical conditions in nearly all electroporated cells including those with low Sox10 levels [25]. While this argues that Myrf induction by Sox10 in the early neural tube is not physiological, it nevertheless shows that Sox10 is capable of inducing Myrf in the CNS in principle. In contrast, Sox10 overexpression failed to induce Myrf in S16 Schwann cells (Figure S4A–F) arguing that even high amounts of Sox10 cannot induce Myrf in this type of peripheral glia.

In case of a direct effector-target gene relationship induction should be mediated by one or several regulatory regions of the *Myrf* gene. To identify such regions we searched for evolutionarily conserved non-coding sequences (ECR) within or near the *Myrf* gene. Using the ECR browser (http://ecrbrowser.dcode.org/) we identified 13 ECR in the interval between the *Myrf* flanking genes *Dagla* and *1810006K21 Rik* (Figure 4F). Most ECR were located in the 11 kb long intron 1. Although originally identified by their conservation among mammals, especially those in intron 1 were conserved down to chicken.

All ECR contained at least one potential binding site for Sox proteins as defined by a perfect or near perfect match (≤1 mismatch) to the 7 bp consensus binding motif 5′-(A/T)(A/T)CAA(A/T)G-3′. However, it is very difficult to predict from the presence of such sites whether a Sox protein actually binds and functions through that site (for review, see [26]). Considering the large number of potential Sox binding sites and the low predictive power of their presence, we decided to assess regulatory potential and Sox10-responsiveness of the ECR in luciferase assays.

We combined each ECR with the minimal *Hsp68* promoter and a luciferase reporter and transfected in rat 33B oligodendroglioma [27]. This cell line expresses Sox10, Sox8 and Olig2 endogenously and thus bears resemblance to oligodendroglia (data not shown). The proximal enhancer modules 1 and 2 from the *Mbp* upstream region [28] served as positive control and induced luciferase levels threefold above the levels obtained with the *Hsp68* promoter alone (Figure 4G). Of all ECR tested, *ECR9* was the only one that reproducibly induced luciferase levels in 33B cells higher than the *Mbp* regulatory region (Figure 4G). *ECR9* also responded strongest to co-transfection of a Sox10-specific shRNA (relative to co-transfection of scrambled shRNA) by losing part of its activity (Figure 4I). It is localized in intron 1 and exhibits conservation in mammals and birds.

*ECR9* similarly increased luciferase activity when combined with the minimal promoter of its own gene (Figure 4H). This promoter had basal activity in 33B cells comparable to the *Hsp68* minimal promoter (data not shown) and was refractory to the presence of a Sox10-specific shRNA (Figure 4J). However, once combined with *ECR9*, induction of the luciferase reporter was not only dramatically increased (Figure 4H), but also became strongly sensitive to the presence of this shRNA (Figure 4J). The strong negative influence of the Sox10-specific shRNA on *ECR9* argues that it is responsive to Sox10.

**Figure 5:**
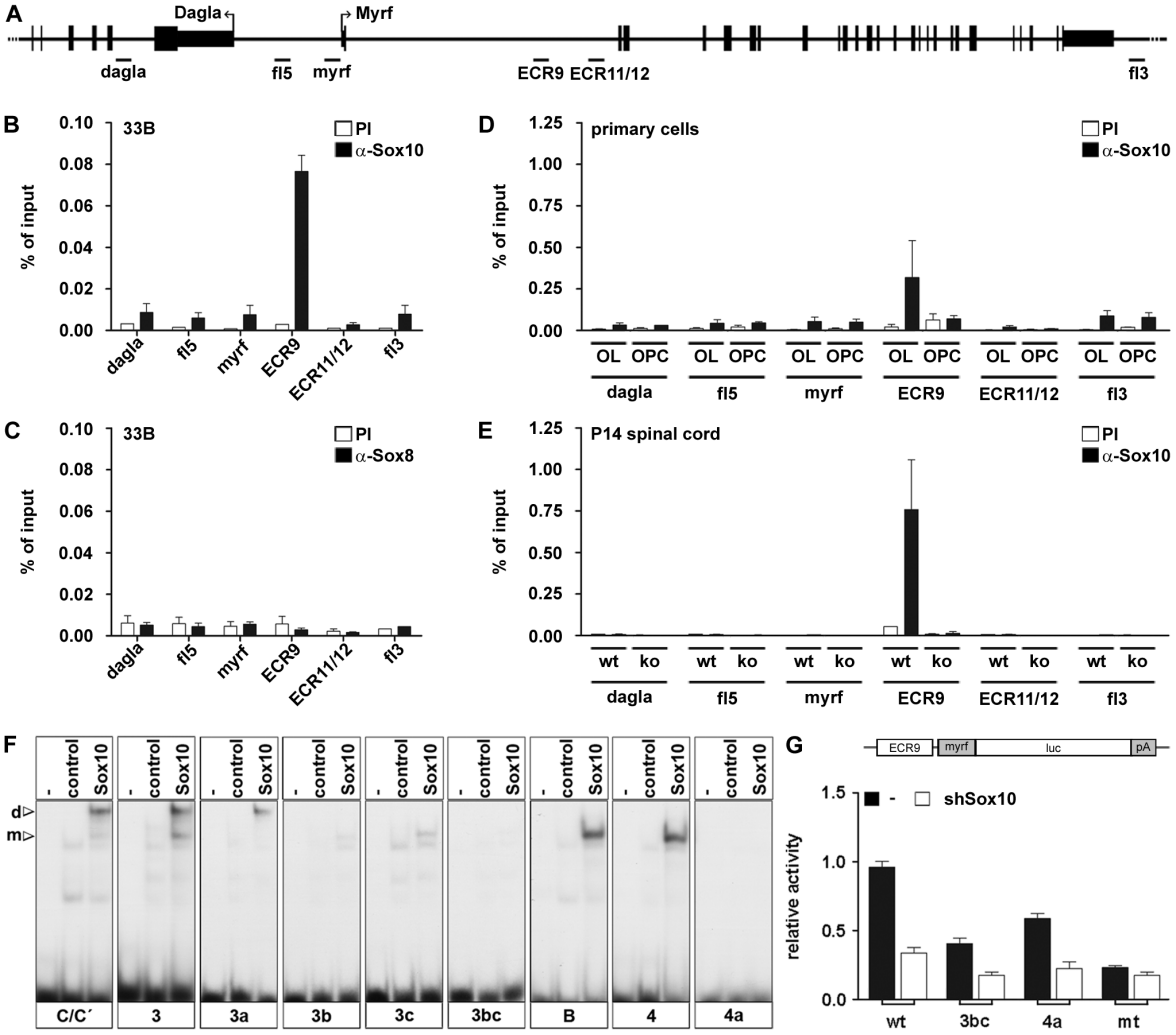
Sox10 binds to intron 1 of the *Myrf* gene both in vivo and in vitro. (**A**) Schematic representation of the location of regions from the *Myrf* locus probed by PCR in ChIP studies including the *Myrf* minimal promoter (myrf), ECR9, ECR11/12 and additional control regions from the 5′ (fl5) and 3′ (3fl) flanking regions of the *Myrf* gene and from within the adjacent *Dagla* gene (dagla). (**B–E**) ChIP was performed on 33B cells (**B,C**), rat primary oligodendroglial cells kept in proliferation medium (OPC) or differentiation medium (OL) (**D**) and P14 spinal cord from wildtype (wt) and *Sox10^ΔCNS^* (ko) mice (**E**) using antibodies directed against Sox10 (α-Sox10) (**B**,**D**,**E**) or Sox8 (α-Sox8) (**C**) and control preimmune serum (PI). Quantitative PCR was applied on the immunoprecipitate. Values for each fragment correspond to the percentage of material precipitated from the input and represent the mean ± SEM of at least 3 biological replicates. (**F**) EMSA was performed with radiolabelled double-stranded oligonucleotides 3 and 4 in wildtype (3, 4) and mutant (3a, 3b, 3c, 3bc, 4a) versions as indicated below the gels. Oligonucleotides were incubated in the absence (−), or presence (control, Sox10) of protein extracts before gel electrophoresis as indicated above the lanes. Extracts were from mock-transfected HEK293 cells (control) or HEK293 cells expressing full length Sox10 (Sox10). Oligonucleotides with site B and site C/C′ from the *Mpz* promoter [29] served as positive control for Sox10 binding and as marker for the mobility of complexes containing either Sox10 monomers (m) or dimers (d). (**G**) *ECR9_myrf-luc* reporter plasmids were co-transfected in wildtype (wt) or mutant (3bc, 4a, mt) version in 33B cells with empty shRNA expression vector or vectors coding for Sox10-specific shRNA. The mt version corresponds to a combination of the 3bc and 4a mutations. Luciferase activities were determined and the activity of the wildtype *ECR9_myrf-luc* reporter in the presence of empty shRNA expression vector was arbitrarily set to 1. All other activities were calculated relative to this value and are presented as mean ± SEM. All experiments were performed three times in quadruplicates.

To verify that Sox10 is bound to *ECR9* in OL we next performed chromatin immunoprecipitation (ChIP) experiments (Figure 5A–E). When chromatin was prepared from 33B cells and subjected to precipitation using antibodies directed against Sox10, substantial amounts of *ECR9* were found (Figure 5B). Precipitates from parallel experiments with preimmune serum contained much less *ECR9*. Such high and specific enrichment was not detected for the minimal *Myrf* promoter, *ECR11* and *ECR12* nor for control fragments located in the 5′ and 3′ flanking regions of the *Myrf* gene or in the adjacent *Dagla* gene (Figure 5A,B). Interestingly, none of the fragments including *ECR9* was enriched when chromatin precipitating antibodies against Sox8 were used for ChIP arguing that *ECR9* is predominantly bound by Sox10 rather than Sox8 in 33B cells (Figure 5C).

Substantial and selective enrichment of *ECR9* was also obtained when ChIP experiments were carried out with Sox10-specific antibodies on primary rat oligodendroglial cells cultured for one day in differentiation medium (Figure 5D). When cells were kept instead in proliferation medium as OPC, no *ECR9* enrichment was observed (Figure 5D). In agreement with these ChIP data from cultured cells, *ECR9* was also preferentially precipitated by Sox10-specific antibodies from chromatin prepared from spinal cord of P14 wildtype mice (Figure 5E). When spinal cord was from *Sox10^ΔCNS^* mice, *ECR9* enrichment was no longer visible (Figure 5E). These results confirm the presence of Sox10 on *ECR9* in an oligodendroglial cell line, in differentiating OL and in vivo.

As ChIP experiments cannot distinguish between direct and indirect binding we searched for Sox10 binding sites within *ECR9*. None of the 11 potential sites (Figure S5A) completely matched the Sox consensus binding site. Some were clustered so that eight oligonucleotides were sufficient to cover all sites in electrophoretic mobility shift assays (EMSA). Site B and site C/C′ from the Myelin Protein Zero gene (*Mpz*) promoter [29] served as positive controls and marked the position of Sox10 monomers and dimers bound to DNA after electrophoresis in native gels (Figure S5B). Surprisingly, only two of the eight oligonucleotides readily bound Sox10. Oligonucleotide 3 preferentially bound Sox10 as a dimer, whereas oligonucleotide 4 preferred the monomer.

To corroborate the binding site on the nucleotide level we introduced mutations. Oligonucleotide 3 contained three potential binding sites (Figure S5C). Mutation of the first site in oligonucleotide 3a did not interfere with binding of a Sox10 dimer, while mutation of the second and third site in oligonucleotides 3b and 3c dramatically decreased overall binding of Sox10 and changed binding preference from dimer to monomer (Figure 5F). Joint mutation of the second and third site in oligonucleotide 3bc abolished Sox10 binding altogether (Figure 5F). This defines the dimer site in *ECR9* as a composite element in which two imperfect Sox consensus sites are separated by four bp and arranged in a head-to-tail fashion (Figure S5C). In oligonucleotide 4, only one potential Sox10 binding site was present and its mutation in oligonucleotide 4a prevented binding completely (Figure 5F). Both sites 3 and 4 exhibit strong sequence conservation in mammals (Figure S5A). Interestingly, they were also bound by Sox8 in vitro despite the fact that *ECR9* occupancy in vivo seems restricted to Sox10 (Figure S5D).

To validate the functional importance of the identified Sox10 binding sites, we introduced the 3bc and 4a mutations into *ECR9* and analyzed the consequences on activity and ability to respond to Sox10. In transiently transfected 33B cells, mutation of either dimer or monomer site led to a significant decrease in the ability of *ECR9* to activate expression of a luciferase reporter under control of the *Myrf* minimal promoter (3bc and 4a in Figure 5G). Joint inactivation of both sites reduced activity even further (mt in Figure 5G). Interestingly, the residual activity of an *ECR9* variant with only one mutated site was still sensitive to the presence of a Sox10-specific shRNA. This sensitivity was lost after simultaneous mutation of monomer and dimer site (Figure 5G).

**Figure 6:**
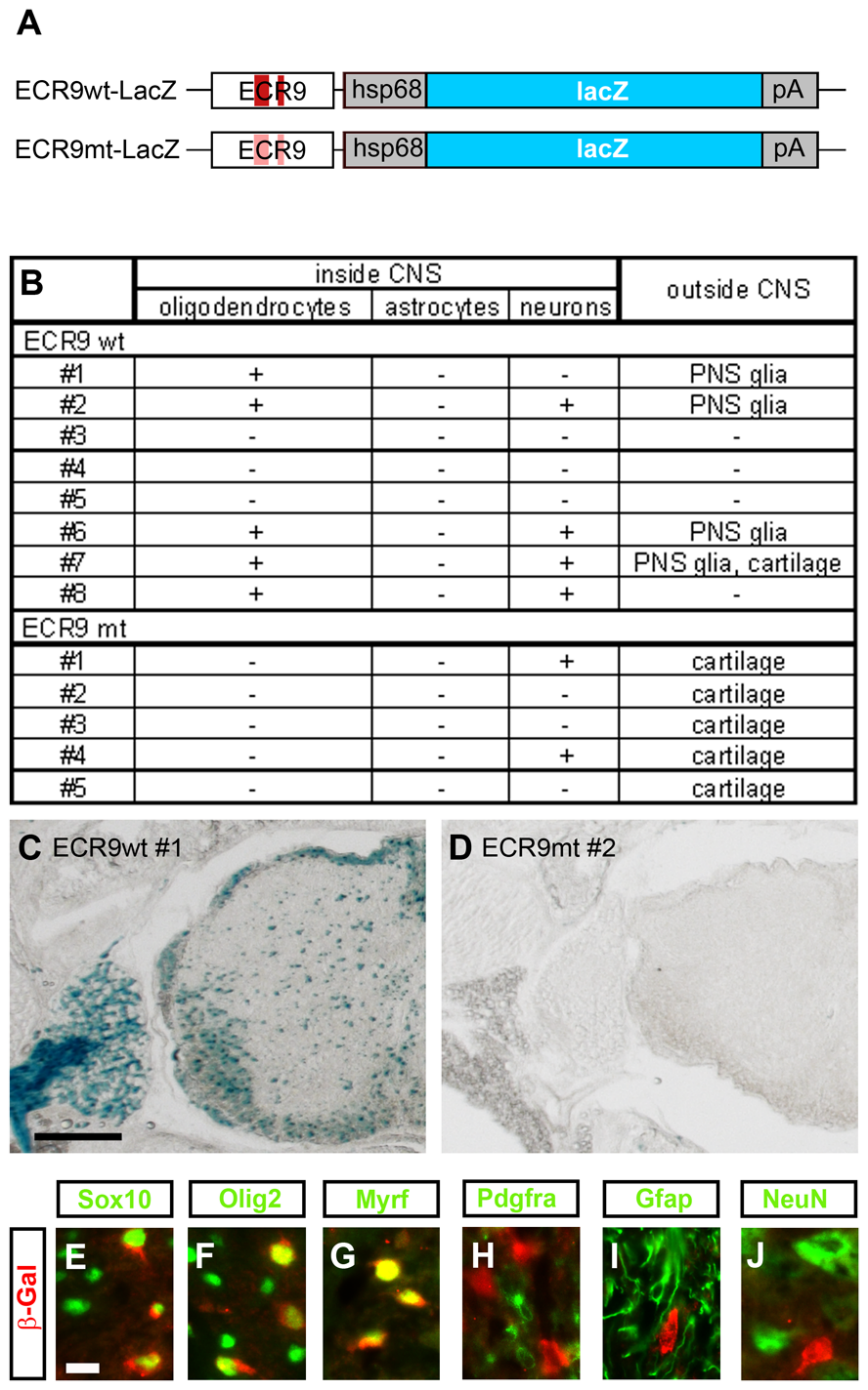
*ECR9* is an OL enhancer in vivo. (**A**) Schematic representation of the transgenic constructs consisting of *ECR9* in wildtype (ECR9wt) or mutant (ECR9mt) version, the minimal *Hsp68* promoter (hsp68), the *lacZ* marker gene (lacZ) and a SV40 polyA signal (pA). Original Sox binding sites are marked in dark red, inactivated ones in light red. (**B**) Summary of lacZ expression patterns in *ECR9wt-lacZ* and *ECR9mt-lacZ* transgenic animals as determined by X-gal staining and immunohistochemistry on transverse sections at P7. (**C,D**) Detection of lacZ expression by X-gal staining of transverse sections from the forelimb level of seven day old pups carrying the *ECR9wt-lacZ* (animal #1) (**C**) or the *ECR9mt-lacZ* (animal #2) (**D**) transgene. Only spinal cord and adjacent tissues are shown. Size bar, 200 µm. (**E–J**) Co-immunohistochemistry was performed on transverse sections of *ECR9wt-lacZ* transgenic animal #1 using antibodies directed against β-galactosidase (in red) in combination with antibodies directed against Sox10 (**E**), Olig2 (**F**), Myrf (**G**), Pdgfra (**H**), Gfap (**I**), and NeuN (**J**) (all in green). Pictures were taken from the dorsal funiculus for **E–I** and from the ventral grey matter for **J**. Size bars correspond to 10 µm.

Both the wildtype *ECR9* and the variant with inactivated monomer and dimer sites were combined with the *Hsp68* minimal promoter and a *lacZ* reporter in a transgenic construct and used for the generation of transgenic animals by pronucleus injection (Figure 6A). The *Hsp68* minimal promoter was used because it has low activity and causes little ectopic expression in transgenic mice when combined with a *lacZ* reporter [30], [31]. For wildtype *ECR9*, eight transgenic animals were obtained and killed for analysis at P7. X-gal staining revealed expression of the *lacZ* transgene in five of these animals (Figure 6B). All five exhibited transgene expression in oligodendroglial cells despite some variability in expression levels and occurrence of additional expression sites (Figure 6B,C). For four animals there was additional *lacZ* expression in a subset of spinal cord neurons and/or in PNS glia. One showed transgene expression in cartilage. None of these sites express Myrf normally [19]. The appearance of ectopic expression sites in this kind of analysis is common and likely results from the fact that additional regulatory sequences of the *Myrf* gene were missing or that the native chromatin context was not preserved in the transgenic construct.

Oligodendroglial expression of the *ECR9wt-lacZ* transgene was restricted in all animals to differentiating cells. This is paradigmatically shown for transgenic animal 1. As evident from X-gal staining, transgene expression in the thoracic spinal cord was strongly enriched in white matter (Figure 6C). Immunohistochemistry furthermore confirmed that β-galactosidase colocalizes with Sox10 and Olig2 as oligodendroglial lineage markers (Figure 6E,F) and with Myrf as a marker of promyelinating and differentiating OL (Figure 6G). In contrast there is virtually no overlap of β-galactosidase with Pdgfra as an OPC marker and Gfap as an astrocytic marker (Figure 6H,I). In this particular transgenic animal there was furthermore no overlap between β-galactosidase and the neuronal marker NeuN (Figure 6J). These data define *ECR9* as an enhancer in differentiating OL.

We also generated five transgenic animals in which the *lacZ* reporter was under control of the mutant *ECR9* (Figure 6B). None of these animals exhibited expression of the *ECR9mt-lacZ* transgene in the oligodendroglial lineage (Figure 6D). Expression was only found in subsets of spinal cord neurons and cartilage. These results argue that the presence of Sox binding sites is essential for *ECR9* activity in OL.

Considering that transgene expression in spinal cord neurons and cartilage was observed similarly in the absence or presence of Sox10 binding sites and that the two cell types express little Sox10 [2], [15], it is unlikely that this ectopic expression is Sox10-dependent. Ectopic expression in peripheral glia may, however, rely on Sox10 as it was only observed in the transgene with intact Sox10 binding sites. This would further support the conclusion that *ECR9* is Sox10-responsive. However, it would also argue that *ECR9* in the context of the transgene is not able to reproduce the differential aspects of Myrf activation in OL as opposed to Schwann cells.

### Myrf Physically and Functionally Interacts with Sox10

**Figure 7:**
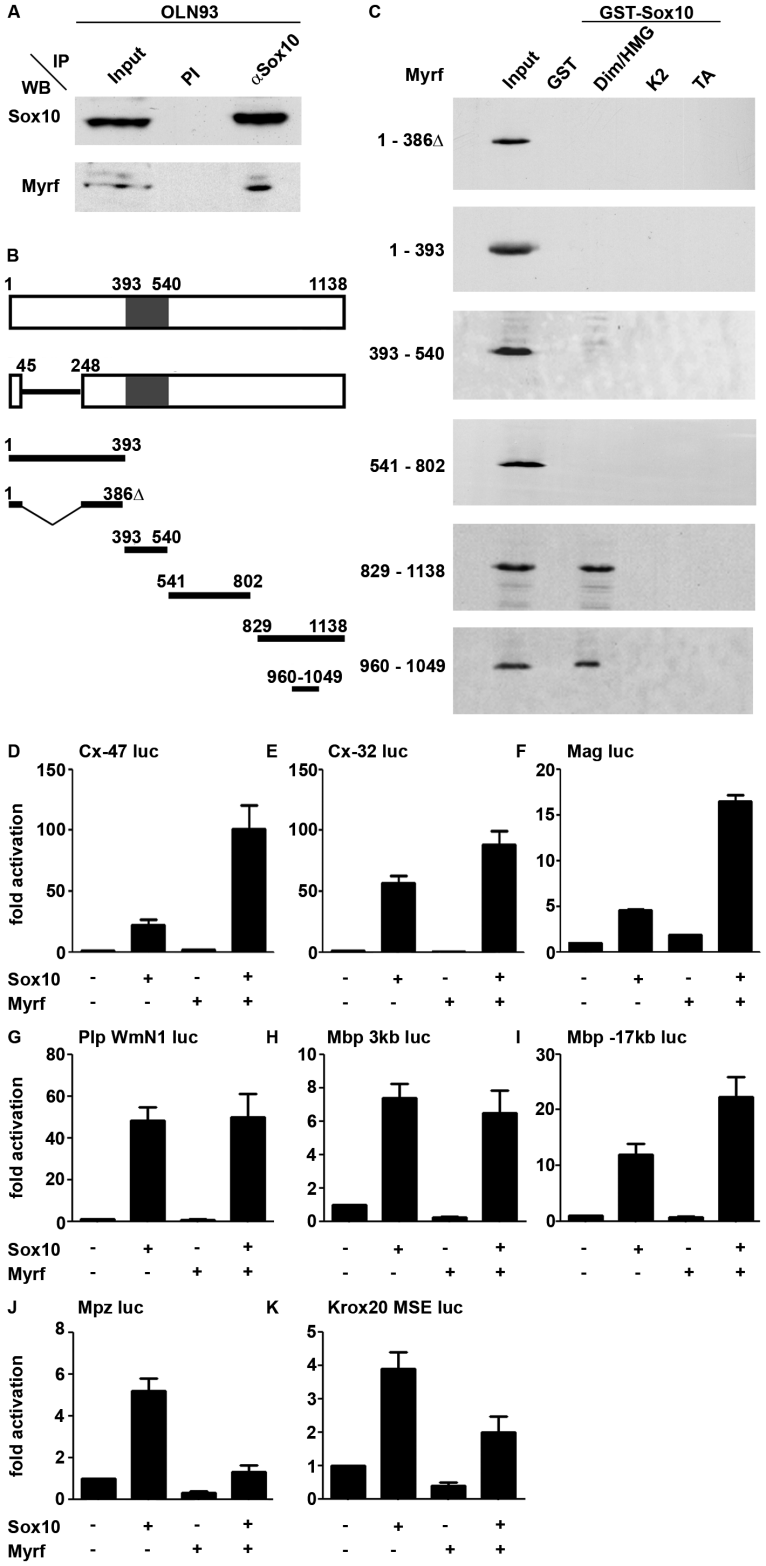
Sox10 and Myrf interact physically and functionally. (**A**) Co-immunoprecipitation (IP) of endogenous Myrf with anti-Sox10 antiserum (αSox10) or preimmune serum (PI) from OLN93 cell extracts. The upper panel shows western blot (WB) detection of Sox10, while the lower panel probes the presence of Myrf in the precipitate using antibodies specifically directed against the carboxyterminal part of the protein. Input corresponds to one tenth of the amount of the protein used in the assay. (**B**) Schematic representation of the Myrf isoform identified by [19] (upper bar, NCBI accession number Q3UR85.2), the splice variant used in this study (lower bar, NCBI accession number AAI57943.1) and various fragments used in interaction studies. Numbers represent amino acid positions. The DNA-binding Ntd80 domain is marked in grey. (**C**) Pulldown assays were performed with Sox10 fragments immobilized as GST-fusions on glutathione sepharose beads and the Myc-tagged Myrf fragments produced in HEK293 cell extracts. Detection of Myrf fragments was by western blot using an antibody directed against the Myc tag. Sox10 regions fused to GST included the dimerization and HMG domains (Dim/HMG), the K2 region and the transactivation domain (TA). (**D–K**) Transient transfections were performed in N2a cells with a luciferase reporter under control of the 727 bp *Cx-47 1b* promoter (**D**), the 416 bp *Cx-32 P2* promoter (**E**), the 626 bp *Mag* promoter (**F**), the 1.2 kb *WmN1 Plp* enhancer (**G**), the 3 kb upstream region of the *Mbp* gene (**H**), a 631 bp conserved region 17 kb upstream of the *Mbp* gene (**I**), the 415 bp *Mpz* promoter (**J**) and the 1.3 kb *MSE Krox20* enhancer (**K**). Empty *pCMV5* expression plasmids (−) or expression plasmids for Sox10 and Myrf were co-transfected as indicated below the bars. Luciferase activities in extracts from transfected cells were determined in at least four experiments each performed in triplicates. The activity obtained for the luciferase reporter in the absence of ectopic transcription factor was arbitrarily set to 1. Fold inductions in the presence of transcription factors were calculated and are presented as mean ± SEM.

Considering that both Sox10 and Myrf are required for terminal differentiation and myelination [16], [19], [20], [23] they may cooperate once Myrf has been induced by Sox10. To address this issue we performed immunoprecipitation experiments with anti-Sox10 antibodies on extracts of the OL-like OLN93 cell line. Sox10 was readily precipitated from OLN93 extracts as shown by western blot (Figure 7A). The precipitate also contained a protein that reacted with an antibody specifically directed against the carboxyterminal region of Myrf arguing that both proteins interact at physiological concentrations.

To confirm this result and map the interacting regions within both proteins, we first generated polypeptides corresponding to defined regions of the major Myrf isoforms (Figure 7B) and analyzed them for their ability to interact with fusion proteins between GST and several conserved Sox10 regions including the dimerization and HMG-domain, the central K2 domain and the carboxyterminal transactivation domain. These GST-pulldown experiments revealed that only the region encompassing dimerization and adjacent HMG domain of Sox10 interacted with Myrf (Figure 7C). For Myrf, interaction with Sox10 was restricted within the carboxyterminal part to an approximately 90 amino acid long segment, 178 to 89 residues away from the carboxyterminus.

To analyze whether physical interaction is paralleled by functional cooperation we performed transient transfections in N2a neuroblastoma cells with luciferase reporters under control of regulatory regions from several genes expressed in differentiating OL (Figure 7D–I). These included the Connexin-47 (*Cx-47*) *1b* promoter, the Connexin-32 (*Cx-32*) *P2* promoter, the Myelin-associated glycoprotein gene (*Mag*) promoter, a 3 kb upstream region of the *Mbp* gene as well as the intronic *WmN1* enhancer of the *Plp* gene and a recently identified Myrf-responsive region 17 kb upstream from the *Mbp* gene [8], [18], [28], [32]–[34]. N2a cells lack endogenous Sox10 and Myrf. All regulatory regions were strongly activated by co-transfected Sox10, while Myrf exerted at most a mild stimulatory effect under the assay conditions. Interestingly, the combination of Sox10 and Myrf led to a dramatic synergistic activation of the *Cx-47 1b*, the *Cx-32 P2* and the *Mag* promoter (Figure 7D–F). Other regulatory regions such as the *Plp WmN1* enhancer and the 3 kb *Mbp* upstream region did not exhibit a synergistic response in the presence of Sox10 and Myrf (Figure 7G,H). However, this does not mean that the corresponding genes are not jointly regulated by the two transcription factors as synergism may be mediated by other regulatory elements of the gene. In accord with such an assumption, the *Mbp* gene contains an evolutionary conserved element 17 kb upstream of the transcriptional start that has recently been identified as Myrf binding [34]. In contrast to the 3 kb upstream region, this region was synergistically activated by both Myrf and Sox10 (Figure 7I). This provides evidence that the two transcription factors cooperate in the activation of at least some myelin genes that occur in OL, partly through known and partly through novel regulatory regions. Preliminary experiments did not yield any evidence, that Myrf is capable of potentiating Sox10 activity on *ECR9* (data not shown). In contrast to the OL-specific regulatory regions, the *Mpz* promoter and the *MSE* enhancer of the *Krox20* gene as Schwann cell-specific regulatory regions [6], [29] were even reduced in their activity by Myrf, and were less Sox10-responsive in the presence of Myrf (Figure 7J,K). Whether this means that Myrf actively represses the expression of Schwann cell genes in OL, remains to be studied in future experiments. In any case, it supports the notion of Myrf as an activator of OL-specific gene expression.

## Discussion

While previous studies on the role of Sox10 in oligodendroglial development were restricted to prenatal stages [16], [17], [23], CNS-specific deletion of a conditional *Sox10* allele for the first time allowed a closer look postnatally. It confirmed our previous assumption that Sox10 is required for terminal OL differentiation and CNS myelination, and proved that these processes are not only delayed, but permanently disrupted in the absence of Sox10. Interestingly, while some residual myelin gene expression was observed, there was no myelin formation.

The residual myelin gene expression in the absence of Sox10 was completely lost when Sox8 was additionally deleted. Again this confirms previous findings that the two closely related Sox proteins participate in OL development and perform partially redundant functions [23]. Previous findings [22] had already established that the impact of Sox10 on OL development is stronger than the impact of Sox8, and that both proteins have similar, but not identical functions. This agrees with our finding that Sox8 allows some minor degree of myelin gene expression in the absence of Sox10, but no myelin formation.

In *Sox10^ΔCNS^* mice Sox8 cannot sustain residual myelin gene expression at uniform levels in all oligodendroglial cells but rather allows fairly normal expression levels in a small subset. These cells do not contain higher amounts of Sox8 than adjacent ones which fail to turn on myelin genes (data not shown) arguing that the residual myelin gene expression in *Sox10^ΔCNS^* mice is not due to stochastic fluctuations of Sox8 amounts in oligodendroglia. It confirms previous findings that a replacement of *Sox10* by *Sox8* coding sequences fails to rescue terminal oligodendroglial differentiation in mice [22], and argues for a heterogeneity among spinal cord OL with the majority mainly relying on Sox10, and a minority being Sox8-dependent.

Our present study also shows for the first time that Sox10 not only acts as a direct regulator of myelin gene expression during OL differentiation [16], but is involved in initiating differentiation by inducing expression of Myrf as the central regulator of OL myelination [19]. Considering that Sox10 is expressed at all stages of oligodendroglial development this induction cannot be triggered by Sox10 alone. Most certainly it additionally requires cell intrinsic or extrinsic signals. These signals either lead to induction of transcription factors that then cooperate with Sox10 during the process, or alter Sox10 activities directly, for instance through posttranslational modifications. A candidate for a cooperating transcription factor is Nkx2.2 which is induced in promyelinating OL [35]. Myrf induction will likely involve one of the Olig proteins as major regulators of OL development as well (for review, see [36]). Putative posttranslational modifications include sumoylation, phosphorylation and acetylation which all occur at multiple sites in Sox10 [37], [38] (data not shown).

Whatever the underlying trigger, Sox10-dependent induction is direct, and at least in part mediated by a conserved intronic enhancer of the *Myrf* gene which we named *ECR9*. We do not want to imply that *ECR9* is the only oligodendroglial enhancer of the *Myrf* gene or that Sox10 acts solely through *ECR9*, but evidence for the involvement of *ECR9* in Sox10-dependent *Myrf* activation is manifold. It is bound in oligodendroglial cell lines and spinal cord by Sox10. It increases expression from minimal promoters in oligodendroglial cells and responds in its activity to the presence of Sox10. It furthermore contains a monomeric and a dimeric binding site for Sox10 that strongly contribute to enhancer activity. Most importantly, *ECR9* is capable of directing reporter gene expression to differentiating OL in transgenic animals, again dependent on the presence of intact Sox binding sites. The fact that *ECR9* exhibited substantial variability in its level of activity in transgenic mice and induced reporter gene expression at sites where Myrf is not normally expressed, may indicate that the enhancer reaches full and specific activity only in its normal genomic context or is only one of several critical enhancers for oligodendroglial Myrf expression.

The fact that ECR9 contains both dimeric and monomeric Sox10 binding sites is not uncommon and has been observed for other Sox10-regulated enhancers (for review, see [26]). Considering that Sox proteins act as architectural proteins [39] and that Sox10 dimers and monomers alter DNA topology differently [40], type and location of each binding site are likely of functional importance.

Once induced, Myrf interacts physically and functionally with Sox10. The physical interaction involves a region in the carboxyterminal part of Myrf. In Sox10, DNA-binding HMG-domain and preceding dimerization domain are involved in the interaction. This region has previously been identified as a hotspot for interactions with other proteins including transcription factors and chromatin remodeling complexes [25], [41]. It is currently unknown to what extent these interactions can occur simultaneously or are mutually exclusive.

There is also functional interaction between the two proteins. At least some regulatory regions from myelin genes are synergistically activated by the combination of Sox10 and Myrf. Others, however, are not, arguing that Sox10 does not necessarily work together with Myrf on every relevant regulatory region. It also argues against a model in which Myrf is simply recruited by its physical interaction with Sox10. Myrf has a DNA-binding domain of its own, and its preferential binding site has recently been determined bioinformatically, but not yet confirmed by EMSA [34]. From all currently available data a scenario appears likely in which both Sox10 and Myrf have to bind separately and directly to the regulatory regions they jointly activate.

Based on our analyses, we come up with a model in which Sox10 induces Myrf in the promyelinating OL and then teams up with Myrf to jointly activate the expression of myelin genes and other essential components of the terminal differentiation and myelination programs. Sox10 thus performs its function during OL development by a positive feed-forward mechanism. A comparable mode of action exists for Sox10 in differentiating Schwann cells of the PNS. Here Sox10 is first needed to induce Oct6 expression [3], [5] before both transcription factors cooperate to induce Krox20 [6]. Krox20 and Sox10 then jointly coordinate terminal differentiation and myelination in Schwann cells [7], [8]. The Sox10-Krox20 regulatory circuit in Schwann cells is thus functionally analogous to the Sox10-Myrf circuit identified here in OL.

This argues that terminal differentiation of PNS and CNS glia and myelination is regulated along similar principles with both processes relying on Sox10 and feed forward mechanisms. However, the exact transcription factors that are induced by Sox10 and then cooperate with Sox10 in the differentiation program are different. In fact, they do not even share structural similarities. The functional equivalent of the Schwann cell differentiation factor Krox20 – a zinc finger protein – is Myrf, a factor with a Ndt80 DNA-binding domain in OL. This divergence of key components argues that both gene regulatory networks have been constructed independently around Sox10 as the common denominator and constitute the result of convergent evolutionary processes. Together with differences in the mode of myelination and with distinct ontogenetic origins of Schwann cells and OL from neural crest and neuroepithelial precursors, respectively, our findings give some support for a model in which the ability to myelinate arose separately in these two glial cell types of the CNS and PNS.

## Materials and Methods

### Plasmids

Several ECR from the *Myrf* genomic region (see Figure 4G) between positions 10,244,770 and 10,207,852 of mouse chromosome 19 (mouse genome version mm10) were amplified by PCR and inserted as SacI/XhoI (*ECR2*; *ECR6*; *ECR7*; *ECR8*; *ECR9*; *ECR10*; *ECR11/12*; *ECR13*), EcoRV/XhoI (*ECR1*), or NheI/XhoI (*ECR4/5*) fragments upstream of the *Hsp68* minimal promoter (positions −104 to +229 relative to the transcriptional start site) into *hsp68-luc*. ECR positions relative to the transcriptional start site of the *Myrf* gene are as follows: *ECR1* (−4022 to −3639), *ECR2* (−3332 to −2869), *ECR3* (−2850 to −2131), *ECR4/5* (−1141 to −144), *ECR6* (+681 to +1376), *ECR7* (+1966 to +2872), *ECR8* (+4536 to +5045), *ECR9* (+7620 to + 8432), *ECR10* (+9081 to +9766), *ECR11/12* (+10089 to +10885) and *ECR13* (+32523 to +32896). Like *hsp68-luc*, the *myrf-luc* reporter plasmid was based on *pGL4.10* (Promega) and contained the *Myrf* minimal promoter (positions −309 to +61). It was used to combine *ECR9* in wildtype (*ECR9wt_myrf-luc*) or mutant versions (*ECR9.3bc_myrf-luc*, *ECR9.4a_myrf-luc*, *ECR9mt_myrf-luc*) with its own gene promoter (Figure 5). Other reporter plasmids used in this study carried the luciferase reporter under control of the *Mbp* 3 kb upstream region [16], the *Cx-47 1b* promoter [18], the *Cx-32 P2* promoter [32], the *Mag* promoter (positions −614 to +12, generated by PCR), the *Mpz* promoter [29], *the Plp WmN1* enhancer (positions +3673 to +4844, generated by PCR), the *Krox20 MSE* enhancer [42] and an evolutionary conserved region 17 kb upstream of the *Mbp* gene (positions −17664 to −17033, generated by PCR), with all enhancers being combined with the β-globin minimal promoter.

The eukaryotic expression plasmids for Sox10 (*pCMV5-Sox10* and *pCAGGS-Sox10-IRES-nls-GFP*) and carboxyterminally truncated Sox8 (pCMV5-Sox8ΔC) have been described [15], [23], [25]. Eukaryotic *pCMV5*-based expression plasmids were also generated for Myrf and several Myrf fragments (see Figure 7B). Myrf fragments were fused to an aminoterminal Myc epitope. For knockdown experiments *pSuper-Neo-GFP* plasmids (Oligoengine) were used that expressed a *Sox10*-specific shRNA (targeted region: 5′-CTGCTGTTCCTTCTTGACCTTGCCC-3′) or a scrambled control shRNA. *ECR9-lacZ* transgenes contained the *ECR9* fragment in wildtype or mutant version (Figure 6A) in front of the *Hsp68* minimal promoter followed by a *lacZ* reporter gene and polyA cassette.

### Transgenic Mice and In Ovo Electroporations

For conditional Sox10 deletion, *Sox10^loxP^* allele [3] and *Brn4::Cre* transgene (bcre-32 line) [21] were combined in *Sox10^ΔCNS^* mice. *Sox10^ΔCNS^ Sox8^lacZ/lacZ^* mice additionally lacked Sox8 [43]. All mice were on a mixed C3H×C57Bl/6J background. Genotyping was performed by PCR [3], [43].

Mice transgenic for the *ECR9-lacZ* transgene in wildtype or mutant form (*ECR9wt-lacZ* and *ECR9mt-lacZ*, see Figure 6A) were obtained by microinjecting a 4.7 kb NotI/XhoI fragment into the male pronucleus of fertilized F1 (C57BL/6×CBA) oocytes according to standard techniques. Pups were killed at P7 and genotyped by PCR using 5′-CCTGGCTTGAGTGTTCTGGT-3′ and 5′-AGTAGCTGTCAGCGTCTGGT-3′ as primers.

In ovo electroporations were carried out on chicken embryos at HH stage 10–11 after injection of expression plasmids into the neural tube. Conditions for electroporation and procedures to obtain, process, and analyze material 24 h post electroporation at HH stage 19–20 were as described [25].

### Tissue Preparation, In Situ Hybridization, Immunohistochemistry, X-gal Staining, Histology, RNA and Chromatin Preparation

Material from staged mouse embryos to P21 mice and from electroporated chicken embryos was processed for light and transmission electron microscopy [3], X-gal staining [16], in situ hybridization with probes specific for *Pdgfra*, *Mbp*, *Plp* or *Myrf*, or for immunohistochemistry using primary antibodies against Sox10, Sox9, Sox8, Olig2, Myrf, Nkx2.2, Pdgfra, CC1, CNPase, Gfap, NeuN, GFP and β-galactosidase. With exception of Myrf and CNPase antibodies, source and working concentration of primary antibodies as well as fluorophore-labelled secondary antibodies were as described [17]. Antibodies against Myrf were generated in rabbit against a bacterially produced peptide spanning amino acids 1–45 and 248–386 in the aminoterminal part of Myrf (NCBI accession number Q3UR85.2). Antibodies against CNPase were from NeoMarker and used in a 1∶250 dilution. Nuclei were counterstained with DAPI. Spinal cord tissue was also used to prepare RNA [3] and sheared cross-linked chromatin [25].

### Quantitative PCR (qPCR) Analysis

RNA samples from mouse spinal cord were reverse transcribed and used to analyze expression levels by qPCR on a Biorad CFX96 Real Time PCR System. The following primer pairs were used: 5′-ACACAAGAACTACCCACTACGG-3′ and 5′-GGGTGTACGAGGTGTCACAA-3′ for *Mbp*, 5′-CCTGTGTCCGTGGTACTGTG-3′ and 5′-TCACACAGGCGGTAGAAGTG-3′ for *Myrf*, 5′-CTGCCTTGCTGATGCTGC GAGA-3′ and 5′-CCTGCCTGGGTCTGCTTGGG-3′ for *Zfp488*, 5′-ACGACAGCAGCGACAACCCC-3′ and 5′-GCTTCCGCTTCTTGCCTGCG-3′ for *Nkx2.2*, 5′-GAAGCAGATGACTGAGCCCGAG-3′ and 5′-CCCGTAGATCTCGCTCA CCAG-3′ for *Olig2*, 5′-ACAGAGACTGAGCGCTGACA-3′ and 5′-CTCGATGGTCTCGTCCTCTC-3′ for *Pdgfra*, 5′-GCCGGTTTCTCTCCGTCGGC-3′ and 5′-GTTGAGAGTGCCGGGGCCTT-3′ for *Zfp191*, 5′-AACGATGATGAGGACC TGAC-3′ and 5′-CAGCTTTCGGGTTCAGATTC-3′ for *Tcf4*, 5′-GGTCACCATGTGGTCCTCGGATG-3′ and 5′-AGGGTCTGAGAGGTCAATGC CAGG-3′ for *Yy1*, 5′-CCCAAGTACCGTGGCGGTGG-3′ and 5′-GCGGCGAAGGCTTTGCTGTG-3′ for *Hes5*, 5′-CCCGGTGGACGACCCGATG-3′ and 5′-CAGATGCCTGCAAGGACAGGATGC-3′ for *Id2*, and 5′-AGGCGGTGAGCCCGGT-3′ and 5′-CGGCCGGGTCAGTGTTGAGC-3′ for *Id4*. Transcript levels were normalized to β-actin.

### Cell Culture, Transient Transfection, Immunocytochemistry, Extract Preparation, EMSA and Reporter Gene Assays

Rat primary oligodendroglial cells were obtained by differential shake-off from mixed glial cultures [44] and grown in Sato proliferation or differentiation medium [45]. Transfection of primary oligodendroglial cells with *pSuper-Neo-GFP*-based expression vectors was by Amaxa nucleofection. 24 h post-transfection, cells were fixed and underwent immunochemical staining using the antibodies described above.

HEK293, 33B, OLN93, S16 and N2a cells were maintained in DMEM containing 10% FCS. S16 cells were used for immunocytochemistry, HEK293 and OLN93 cells for extract preparation, and N2A and 33B cells for luciferase assays.

In case cells were transfected, extract preparation was 48 h post-transfection [18]. HEK293 cells were transfected using polyethylenimine (PEI) and 10 µg *pCMV5*-based Sox10 expression plasmid per 100-mm plate. After verification of ectopic expression by western blotting, EMSA were performed [46] using ^32^P-labeled 25–26 bp double-stranded oligonucleotides containing putative Sox10 binding sites.

For luciferase reporter gene assays, 33B and N2a cells were used. 33B cells were transfected with PEI on 24-well tissue culture plates using 500 ng of luciferase reporter and 100 ng of *pCMV5-* or *pSuper-Neo-GFP*-based expression vectors. N2a cells were transfected with Superfect (Qiagen) on 35 mm plates using 1 µg of luciferase reporter and 500 ng of *pCMV5*-based expression plasmids. Cells were generally harvested 48 h post-transfection except for knockdown experiments where analysis took place 72 h post-transfection. Luciferase activity was determined in the presence of luciferin substrate by detection of chemiluminescence.

### GST Pulldown Assay, Co-immunoprecipitation and Western Blotting

For GST pulldown assays, Myc-tagged Myrf fragments were produced in transfected HEK293 cells and used as whole cell extracts. Sox10 fragments were made in *E.coli* BL21 DE3 pLysS bacteria as GST fusion proteins and purified by affinity chromatography on glutathione-sepharose 4B beads. The bead-bound GST or GST fusion proteins were incubated with HEK293 cell extracts for 2 hours. For co-immunoprecipitation, OLN93 cell extracts were incubated overnight at 4°C with rabbit antiserum against Sox10 (1∶3000 dilution, [47]) or control preimmune serum, and protein A sepharose CL-4B beads (GE Healthcare).

Bead-bound proteins in GST-pulldown assays and co-immunoprecipitations were eluted after repeated cycles of centrifugation and washing, and size fractionated on polyacrylamide-SDS gels. Detection was by western blot using mouse antibodies directed against the Myc epitope tag (1∶10000 dilution, Novagen), and rabbit antisera against Sox10 (1∶3000 dilution), or the carboxyterminal part of Myrf (1∶1000 dilution, generated against amino acids 852–1007 according to NCBI accession number Q3UR85.2).

### ChIP

Chromatin was prepared from 33B cells, primary oligodendroglial cells and spinal cord from P14 wildtype and *Sox10^ΔCNS^* mice after trypsinization, cross-linking of endogenous proteins to DNA and shearing [25]. Immunoprecipitation was overnight at 4°C using Sox10-specific and Sox8-specific antibodies or control immunoglobulins in the presence of pretreated protein A sepharose CL-4B beads. After washing, crosslink reversal, proteinase K treatment, phenol/chloroform extraction and ethanol precipitation, the amount of DNA from input and precipitated chromatin was quantified by qPCR using the Biorad CFX96 Real Time PCR system [25]. To detect various regions of the *Myrf* gene the following primer pairs were used: 5′-GGGTCTGGTATTCGTAGGTC-3′ and 5′-GTGTTCTCTGTACCTCTTGG-3′ to amplify positions -9415 to -9602 relative to the transcriptional start site of the *Myrf* gene in rat and 5′- GTAAGTGGGTCTCTGTGTGC-3′ and 5′- GTGGGTTCAGAATCTGCATAG -3′ to amplify positions -9392 to -9581 in mouse (dagla), 5′- GAGTCCCAGAGTCTAGTAGG -3′ and 5′- CTGGTGTCCTGCCATGTCAG -3′ to amplify positions -3973 to -4152 in rat and 5′- GACAATGTGAATACCCAGTC -3′ and 5′- CCTGATGAACTGACAAGATG -3′ to amplify positions -2682 to -2891 in mouse (fl5), 5′- GTATTGTGCTAGGCCTGCAC -3′ and 5′- GGCAGAAGAAGGCAGTTCTC -3′ to amplify positions - 481 to - 654 in rat and 5′- GCCTTGCCTTAAAGTCTGTG -3′ and 5′- CTCCCTAACAAAGACCTGCC -3′ to amplify positions - 149 to - 364 in mouse (myrf), 5′- CACGTGGCTGACGGGATTTC -3′ and 5′- CCACAGCTGTGGCTGCTGGC -3′ to amplify positions +7969 to +8136 in rat and positions +7586 to +8025 in mouse (ECR9), 5′- GCATTTGAAGAATGCTGAGCC -3′ and 5′- GACAGACTGACCATGTACAGC -3′ to amplify positions +10463 to +10666 in rat and 5′- GTCCAGGGCTTCTGATCATG -3′ and 5′- GGTCCTTCCTGCCTCAGTGG -3′ to amplify positions +10677 to +10885 in mouse (ECR11/12), 5′- CTATGCACACCTCTTGCCAC -3′ and 5′- GAGCCCATTGTTCTAAGAGAC -3′ to amplify positions +35839 to +36087 in rat and 5′- CCATGCCTACTTCTGCGTTG -3′ and 5′- GGAATTCCTTGCCACCACAC -3′ to amplify positions +32539 to +32737 in (fl3).

### Ethics Statement

Mice experiments were in accord with animal welfare laws and approved by the responsible local committees and government bodies.

## Supporting Information

Figure S1 Efficiency of CNS-specific Sox10 deletion and consequences on Sox9 and Sox8 expression. (**A–R**) The expression of Sox10 (**A–H**), Sox8 (**I–P**) and Sox9 (**Q,R**) was analyzed by immunohistochemistry with specific antibodies from P3 (**A,E,I,M,Q,R**) via P7 (**B,F,J,N**) and P14 (**C,G,K,O**) to P21 (**D,H,L,P**) in transverse spinal cord sections from the forelimb region of wildtype (wt) (**A–D,I–L,Q**) or *Sox10^ΔCNS^* (ko) (**E–H,M–P,R**) embryos. Scale bars, 100 µm. (**S–U**) The number of cells positive for Sox10 (**S**), Sox9 (**T**), and Sox8 (**U**) was quantified during the first three postnatal weeks in spinal cord sections of *Sox10^ΔCNS^* (white bars) and wildtype (black bars) pups. For quantifications, at least 9 separate sections from the forelimb region of 3 independent specimens were counted for each age and genotype. Data are presented as mean ± SEM for biological replicates. Differences to the wildtype were statistically significant between wildtype and mutant for Sox10-positive cells and for Sox8-positive cells from P7 onwards as determined by the Student's *t* test (***, P≤0.001).
(TIF)

Figure S2 Consequences of CNS-specific Sox10 deletion on oligodendroglial cell numbers. (**A**,**B**) *Pdgfra*-positive OPC were visualized by in situ hybridization on 18.5 dpc in transverse spinal cord sections from the forelimb region of wildtype (wt) (**A**) and *Sox10^ΔCNS^* (ko) (**B**) embryos. Scale bar, 200 µm. (**C**) Using stained spinal cord sections from in situ hybridizations, the number of *Pdgfra*-positive OPC was quantified in both genotypes at 18.5 dpc. (**D**) The number of Olig2-positive oligodendroglial cells was quantified during the first three postnatal weeks in spinal cord sections of *Sox10^ΔCNS^* (white bars) and wildtype (black bars) pups. For quantifications in C and D, at least 9 separate sections from the forelimb region of 3 independent specimens were counted for each age and genotype. Data are presented as mean ± SEM for biological replicates. Differences to the wildtype were statistically significant for oligodendroglial cell numbers between wildtype and mutant from P7 onwards as determined by the Student's *t* test (**, P≤0.01; ***, P≤0.001). (**E–L**) Olig2 immunoreactivity was detected at P3 (**E,I**), P7 (**F,J**), P14 (**G,K**) and P21 (**H,L**) in transverse spinal cord sections from the forelimb region of wildtype (**E–H**) or *Sox10^ΔCNS^* (**I–L**) embryos. Scale bar, 100 µm.
(TIF)

Figure S3 Consequences of CNS-specific Sox10 deletion on expression levels of stage-specific oligodendroglial markers. (**A**) Immunohistochemistry was performed on transverse spinal cord sections from the forelimb region of wildtype (wt) or *Sox10^ΔCNS^* (ko) mice at P7 and P14 using antibodies directed against Gpr17 (red) as a marker of the promyelinating stage in combination with Sox8 (green) as an OL marker. Magnifications from the ventral horn region are shown. Scale bar, 50 µm. (**B,C**) Quantitative RT-PCR was performed on cDNA prepared from spinal cord of wildtype (wt, black bars) and *Sox10^ΔCNS^* (ko, white bars) mice at P7 (**B**) and P14 (**C**) using primers directed against *Mbp*, *Myrf*, *Zfp488*, *Nkx2.2*, *Olig2*, *Pdgfra*, *Zfp191*, *Yy1*, *Tcf4*, *Hes5*, *Id2* and *Id4* transcripts. After normalization to β-actin transcript levels in the wildtype were arbitrarily set to 1. Experiments were repeated at least three times with material from three independent spinal cord preparations for each genotype. Differences to the wildtype were statistically significant as indicated (Student's *t* test; *, P≤0.05; **, P≤0.01).
(TIF)

Figure S4 Sox10 cannot activate Myrf in Schwann cells. (**A–F**) S16 Schwann cells were transfected with expression vectors for GFP (**A–C**) or a combination of GFP and Sox10 (**D–F**). Two days later transfected cells were identified by GFP expression (**A**,**D**; in green) and analyzed for their expression of Myrf (**B**,**E**; in red) as indicated. Nuclei were visualized by a DAPI counterstain in the merged pictures (**C**,**F**; in blue). Scale bar, 75 µm.
(TIF)

Figure S5 Sox10 and Sox8 recognize a monomer and a dimer site in *ECR9* in vitro. (**A**) The sequence of mouse *ECR9* is shown. Asterisks below the sequence indicate positions that are fully conserved between mouse and human. Putative Sox10 binding sites are marked by a bar above the sequence. Oligonucleotide sequences 1–8 are highlighted by grey boxes with oligonucleotide number at the 3′ end of the sequence. (**B**) EMSA was performed with radiolabelled double-stranded oligonucleotides 1–8 from *ECR9* as indicated below the gels. Oligonucleotides were incubated in the absence (−), or presence (control, Sox10) of protein extracts before gel electrophoresis as indicated above the lanes. Extracts were from mock-transfected HEK293 cells (control) or HEK293 cells expressing full length Sox10 (Sox10). Oligonucleotides with site B and site C/C′ from the *Mpz* promoter [29] served as positive control for Sox10 binding and as marker for the mobility of complexes containing either Sox10 monomers (m) or dimers (d). (**C**) The sequence of oligonucleotides 3 and 4 are shown. Mutant versions 3a, 3b, 3a,b, 4a helped to define the exact location of the Sox10 binding sites. Potential binding sites are indicated by bars above the sequence. Mutated nucleotides are in small letters. (**D**) Additional EMSA were performed with radiolabelled sites B, C/C′, 3 and 4 in wildtype (3, 4) and mutant 3bc, 4a) versions using extracts from HEK293 cells expressing a carboxyterminally truncated Sox8 (Sox8) and the corresponding controls (see **B**).
(TIF)
