# Supplementary figures and images for: The Transcription Factors Sox10 and Myrf Define an Essential Regulatory Network Module in Differentiating Oligodendrocytes
Source: PLoS Genet. 2013 Oct 31;9(10):e1003907. doi: 10.1371/journal.pgen.1003907 (PMC3814293; doi:10.1371/journal.pgen.1003907)

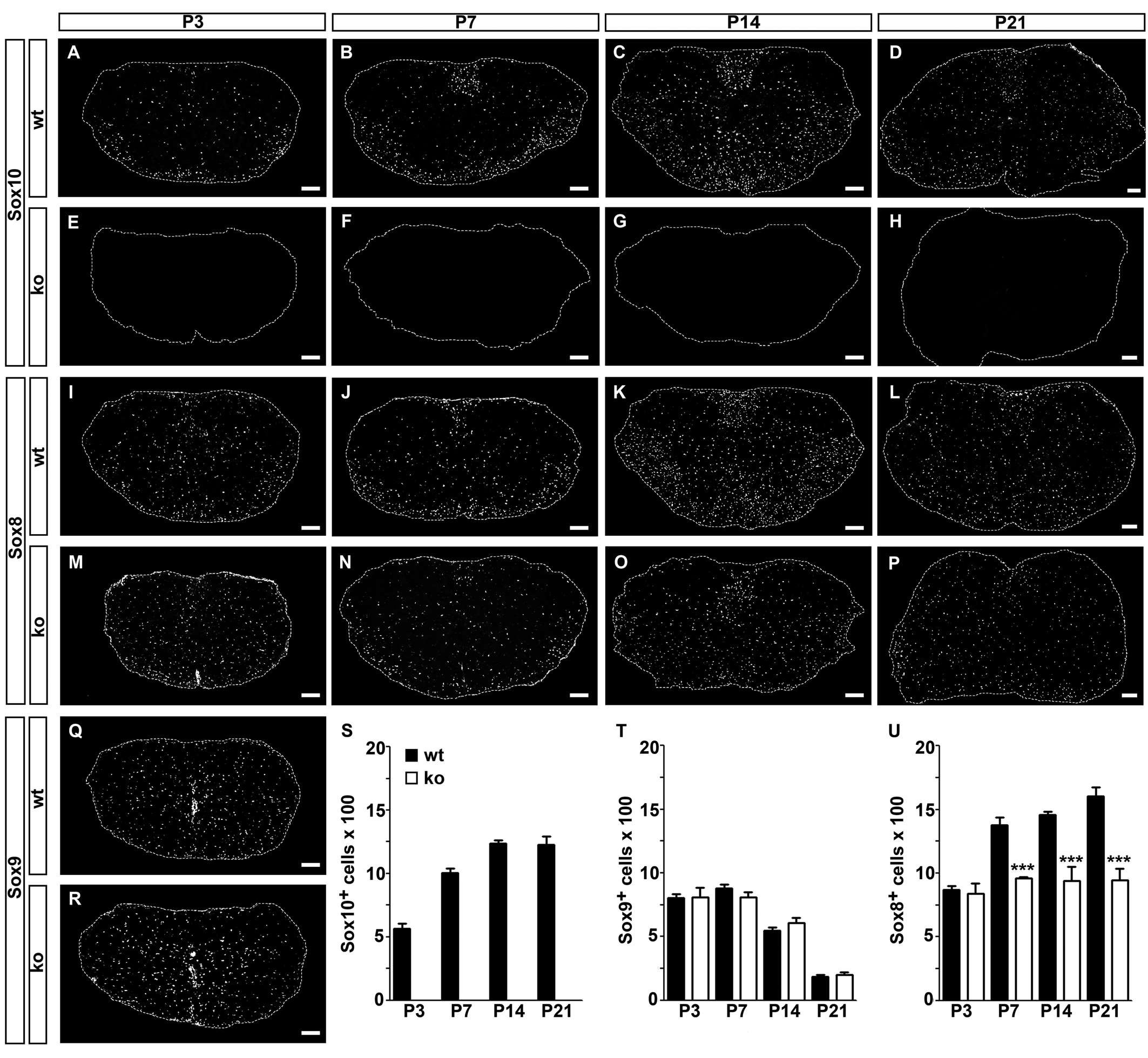

Supplement: Figure S1 — Efficiency of CNS-specific Sox10 deletion and consequences on Sox9 and Sox8 expression. (A–R) The expression of Sox10 (A–H), Sox8 (I–P) and Sox9 (Q,R) was analyzed by immunohistochemistry with specific antibodies from P3 (A,E,I,M,Q,R) via P7 (B,F,J,N) and P14 (C,G,K,O) to P21 (D,H,L,P) in transverse spinal cord sections from the forelimb region of wildtype (wt) (A–D,I–L,Q) or Sox10ΔCNS (ko) (E–H,M–P,R) embryos. Scale bars, 100 µm. (S–U) The number of cells positive for Sox10 (S), Sox9 (T), and Sox8 (U) was quantified during the first three postnatal weeks in spinal cord sections of Sox10ΔCNS (white bars) and wildtype (black bars) pups. For quantifications, at least 9 separate sections from the forelimb region of 3 independent specimens were counted for each age and genotype. Data are presented as mean ± SEM for biological replicates. Differences to the wildtype were statistically significant between wildtype and mutant for Sox10-positive cells and for Sox8-positive cells from P7 onwards as determined by the Student's t test (***, P≤0.001). (TIF) [file pgen.1003907.s001.tif]

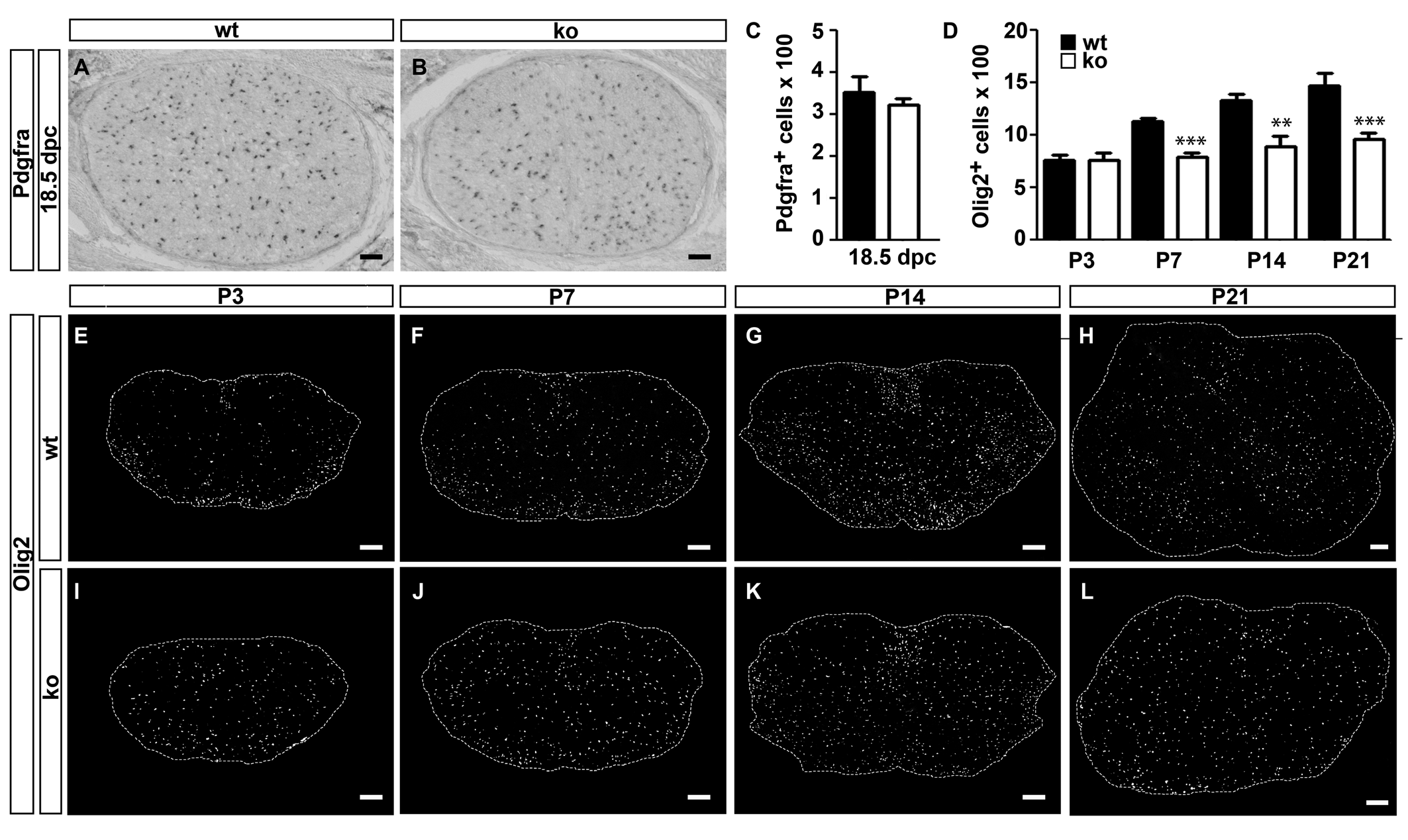

Supplement: Figure S2 — Consequences of CNS-specific Sox10 deletion on oligodendroglial cell numbers. (A,B) Pdgfra-positive OPC were visualized by in situ hybridization on 18.5 dpc in transverse spinal cord sections from the forelimb region of wildtype (wt) (A) and Sox10ΔCNS (ko) (B) embryos. Scale bar, 200 µm. (C) Using stained spinal cord sections from in situ hybridizations, the number of Pdgfra-positive OPC was quantified in both genotypes at 18.5 dpc. (D) The number of Olig2-positive oligodendroglial cells was quantified during the first three postnatal weeks in spinal cord sections of Sox10ΔCNS (white bars) and wildtype (black bars) pups. For quantifications in C and D, at least 9 separate sections from the forelimb region of 3 independent specimens were counted for each age and genotype. Data are presented as mean ± SEM for biological replicates. Differences to the wildtype were statistically significant for oligodendroglial cell numbers between wildtype and mutant from P7 onwards as determined by the Student's t test (**, P≤0.01; ***, P≤0.001). (E–L) Olig2 immunoreactivity was detected at P3 (E,I), P7 (F,J), P14 (G,K) and P21 (H,L) in transverse spinal cord sections from the forelimb region of wildtype (E–H) or Sox10ΔCNS (I–L) embryos. Scale bar, 100 µm. (TIF) [file pgen.1003907.s002.tif]

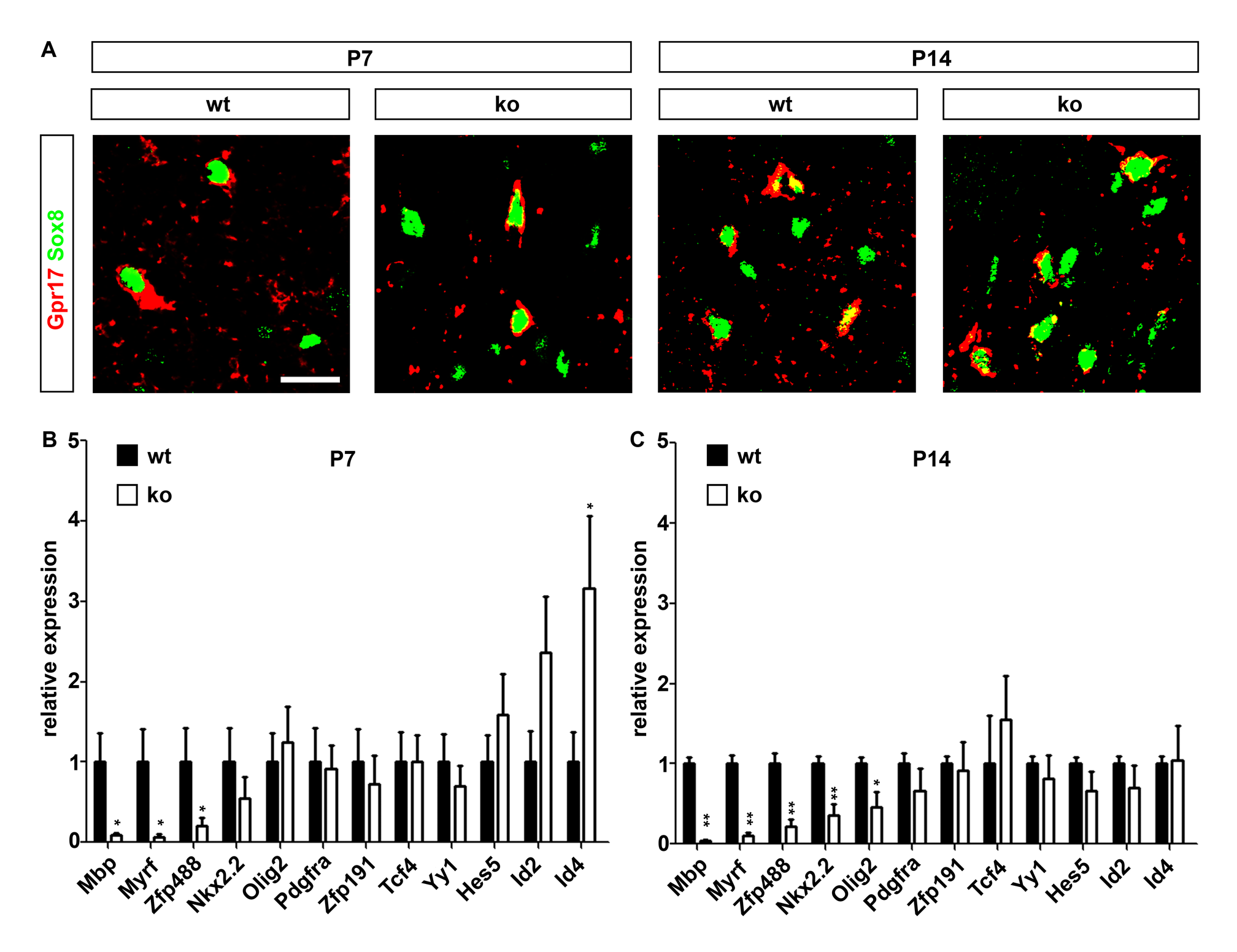

Supplement: Figure S3 — Consequences of CNS-specific Sox10 deletion on expression levels of stage-specific oligodendroglial markers. (A) Immunohistochemistry was performed on transverse spinal cord sections from the forelimb region of wildtype (wt) or Sox10ΔCNS (ko) mice at P7 and P14 using antibodies directed against Gpr17 (red) as a marker of the promyelinating stage in combination with Sox8 (green) as an OL marker. Magnifications from the ventral horn region are shown. Scale bar, 50 µm. (B,C) Quantitative RT-PCR was performed on cDNA prepared from spinal cord of wildtype (wt, black bars) and Sox10ΔCNS (ko, white bars) mice at P7 (B) and P14 (C) using primers directed against Mbp, Myrf, Zfp488, Nkx2.2, Olig2, Pdgfra, Zfp191, Yy1, Tcf4, Hes5, Id2 and Id4 transcripts. After normalization to β-actin transcript levels in the wildtype were arbitrarily set to 1. Experiments were repeated at least three times with material from three independent spinal cord preparations for each genotype. Differences to the wildtype were statistically significant as indicated (Student's t test; *, P≤0.05; **, P≤0.01). (TIF) [file pgen.1003907.s003.tif]

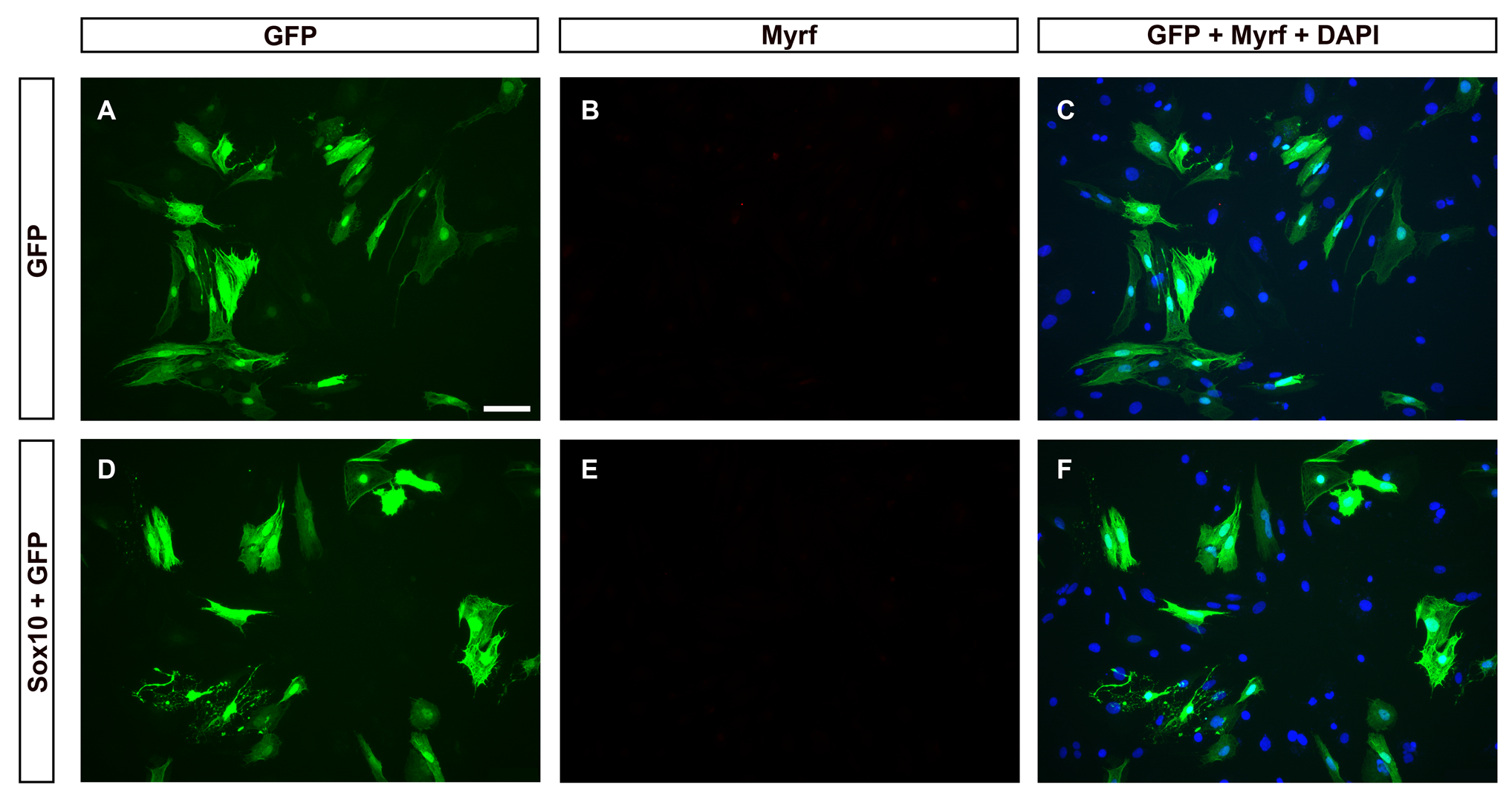

Supplement: Figure S4 — Sox10 cannot activate Myrf in Schwann cells. (A–F) S16 Schwann cells were transfected with expression vectors for GFP (A–C) or a combination of GFP and Sox10 (D–F). Two days later transfected cells were identified by GFP expression (A,D; in green) and analyzed for their expression of Myrf (B,E; in red) as indicated. Nuclei were visualized by a DAPI counterstain in the merged pictures (C,F; in blue). Scale bar, 75 µm. (TIF) [file pgen.1003907.s004.tif]

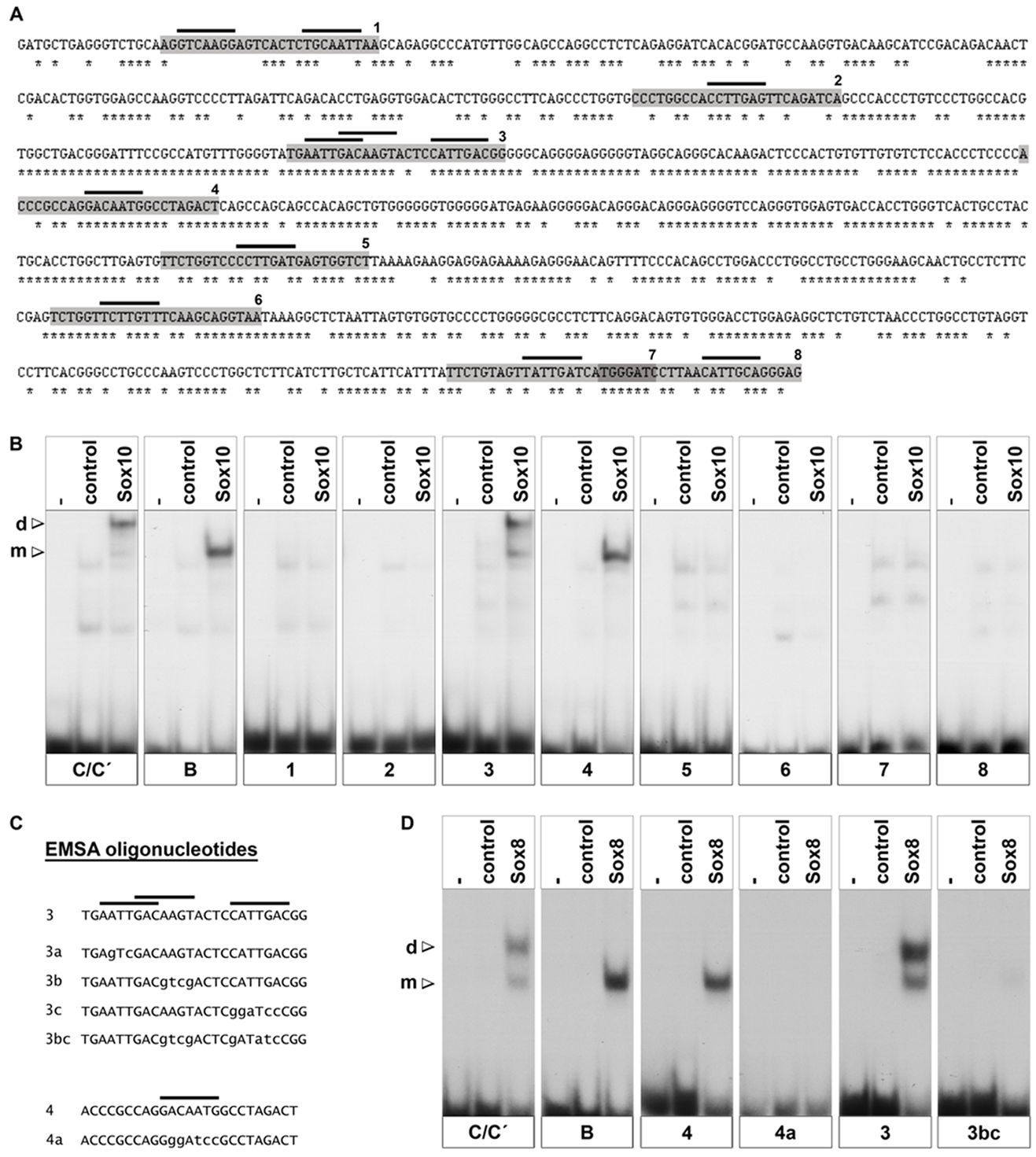

Supplement: Figure S5 — Sox10 and Sox8 recognize a monomer and a dimer site in ECR9 in vitro. (A) The sequence of mouse ECR9 is shown. Asterisks below the sequence indicate positions that are fully conserved between mouse and human. Putative Sox10 binding sites are marked by a bar above the sequence. Oligonucleotide sequences 1–8 are highlighted by grey boxes with oligonucleotide number at the 3′ end of the sequence. (B) EMSA was performed with radiolabelled double-stranded oligonucleotides 1–8 from ECR9 as indicated below the gels. Oligonucleotides were incubated in the absence (−), or presence (control, Sox10) of protein extracts before gel electrophoresis as indicated above the lanes. Extracts were from mock-transfected HEK293 cells (control) or HEK293 cells expressing full length Sox10 (Sox10). Oligonucleotides with site B and site C/C′ from the Mpz promoter [29] served as positive control for Sox10 binding and as marker for the mobility of complexes containing either Sox10 monomers (m) or dimers (d). (C) The sequence of oligonucleotides 3 and 4 are shown. Mutant versions 3a, 3b, 3a,b, 4a helped to define the exact location of the Sox10 binding sites. Potential binding sites are indicated by bars above the sequence. Mutated nucleotides are in small letters. (D) Additional EMSA were performed with radiolabelled sites B, C/C′, 3 and 4 in wildtype (3, 4) and mutant 3bc, 4a) versions using extracts from HEK293 cells expressing a carboxyterminally truncated Sox8 (Sox8) and the corresponding controls (see B). (TIF) [file pgen.1003907.s005.tif]
